# Allele-dependent interaction of LRRK2 and NOD2 in leprosy

**DOI:** 10.1371/journal.ppat.1011260

**Published:** 2023-03-27

**Authors:** Monica Dallmann-Sauer, Yong Zhong Xu, Ana Lúcia França da Costa, Shao Tao, Tiago Araujo Gomes, Rhana Berto da Silva Prata, Wilian Correa-Macedo, Jérémy Manry, Alexandre Alcaïs, Laurent Abel, Aurélie Cobat, Vinicius M. Fava, Roberta Olmo Pinheiro, Flavio Alves Lara, Christian M. Probst, Marcelo T. Mira, Erwin Schurr

**Affiliations:** 1 Program in Infectious Diseases and Immunity in Global Health, The Research Institute of the McGill University Health Centre; Montreal, Canada; 2 McGill International TB Centre, McGill University; Montreal, Canada; 3 Departments of Human Genetics and Medicine, Faculty of Medicine and Health Science, McGill University; Montreal, Canada; 4 Graduate Program in Health Sciences, School of Medicine and Life Sciences, Pontifícia Universidade Católica do Paraná; Curitiba, Brazil; 5 Department of Specialized Medicine, Health Sciences Center, Federal University of Piauí; Teresina, Brazil; 6 Division of Experimental Medicine, Faculty of Medicine, McGill University; Montreal, Canada; 7 The Translational Research in Respiratory Diseases Program, The Research Institute of the McGill University Health Centre; Montreal, Canada; 8 Laboratory of Cellular Microbiology, Oswaldo Cruz Institute, Oswaldo Cruz Foundation; Rio de Janeiro, Brazil; 9 Leprosy Laboratory, Oswaldo Cruz Institute, Oswaldo Cruz Foundation; Rio de Janeiro, Brazil; 10 Department of Biochemistry, Faculty of Medicine and Health Science, McGill University; Montreal, Canada; 11 Laboratory of Human Genetics of Infectious Diseases, Necker Branch, Institut National de la Santé et de la Recherche Médicale U.1163, Paris, France; 12 Université Paris Cité, Imagine Institute, Paris, France; 13 St. Giles Laboratory of Human Genetics of Infectious Diseases, Rockefeller Branch, Rockefeller University, New York, United States of America; 14 Laboratory of Systems and Molecular Biology of Trypanosomatids, Instituto Carlos Chagas; FIOCRUZ, Curitiba, Brazil; University of Washington, UNITED STATES

## Abstract

Leprosy, caused by *Mycobacterium leprae*, rarely affects children younger than 5 years. Here, we studied a multiplex leprosy family that included monozygotic twins aged 22 months suffering from paucibacillary leprosy. Whole genome sequencing identified three amino acid mutations previously associated with Crohn’s disease and Parkinson’s disease as candidate variants for early onset leprosy: *LRRK2* N551K, R1398H and *NOD2* R702W. In genome-edited macrophages, we demonstrated that cells expressing the LRRK2 mutations displayed reduced apoptosis activity following mycobacterial challenge independently of NOD2. However, employing co-immunoprecipitation and confocal microscopy we showed that LRRK2 and NOD2 proteins interacted in RAW cells and monocyte-derived macrophages, and that this interaction was substantially reduced for the NOD2 R702W mutation. Moreover, we observed a joint effect of LRRK2 and NOD2 variants on Bacillus Calmette-Guérin (BCG)-induced respiratory burst, NF-κB activation and cytokine/chemokine secretion with a strong impact for the genotypes found in the twins consistent with a role of the identified mutations in the development of early onset leprosy.

## Author summary

Children younger than 5-years old are rarely affected by leprosy, a chronic infectious disease of the skin and peripheral nerves caused by *Mycobacterium leprae*. Here, we report a case of a family with three generation of leprosy cases including a rare pair of identical twins of less than two years of age. To investigate if genetic factors in the twins may explain the early disease onset, we conducted a whole genome sequence analysis of family members. We found three amino acid changes in LRRK2 and NOD2 as candidate susceptibility variants for early onset leprosy. In follow-up functional assays, we demonstrated that LRRK2 and NOD2 proteins physically interact in macrophages and that this interaction was strongly impacted by the NOD2 variant. Furthermore, we showed a joint effect of LRRK2 and NOD2 variants that results in a reduced antimycobacterial response in these cells. Interestingly, the variants in LRRK2 and NOD2 found in the twins had previously been identified as genetic modulators for risk of Parkinson’s and Crohn’s diseases with the two LRRK2 variants showing antagonistic pleiotropy. This suggests pleiotropic effects of the NOD2 and LRRK2 variants across neurodegenerative, inflammatory and infectious diseases.

## Introduction

Leprosy is a disease of the skin and peripheral nerves that is caused by infection with *Mycobacterium leprae* or *M*. *lepromatosis*. The mode of transmission of leprosy is not completely understood. Evidence suggests zoonotic transmission from armadillos in isolated leprosy cases, and experimental models have presented Reduviidae bugs and ticks as potential vectors [1–3]. However, the most likely path is via human-to-human transmission by close and prolonged contact with an untreated person infected with *M*. *leprae* [4]. Although effective antimicrobial drugs are available, in 2019 over 200,000 new cases of leprosy were detected globally [5]. The majority of leprosy cases are diagnosed in early adulthood (age 20–40 years) and less than 10% of global cases fall below the 15-year age group [6]). Even under conditions of high transmission, only 1% of cases are in the 1–4 years age group with cases younger than 2 years being exceedingly rare [6]. This age distribution of cases suggests that prolonged exposure to *M*. *leprae* or a long incubation period are necessary to result in clinical disease for the majority of exposed persons.

The first genome-wide association study (GWAS) of leprosy identified a striking overlap of genetic risk factors for leprosy and Crohn’s disease (CD), an inflammatory bowel disease (IBD) characterized by a chronic relapsing intestinal inflammation [7,8]. When the comparison was extended to the level of risk variants for IBD and excessive inflammatory episodes in leprosy, termed type-1 reactions (T1R), it became apparent that a majority of risk variants were shared between T1R and IBD while a smaller proportion of risk variants were shared between leprosy *per se* and IBD [9]. GWAS for common variants and rare coding region mutations successfully identified numerous leprosy susceptibility genes [10]. Although there is strong experimental evidence that age-at-onset is an important covariate for genetic effects, most studies in leprosy–including the GWAS studies–were focused on adult leprosy patients [11]. This is in striking difference to other mycobacterial diseases, including tuberculosis (TB), where the focus on early onset patients with strong phenotypes has been extraordinarily successful for the identification of susceptibility genes and a better understanding of disease pathogenesis [12–14].

In the present study, we followed the proven strategy of focusing on genetic factors in early onset patients. We identified a multiplex leprosy family that included monozygotic twins who had developed leprosy before the age of two years, which is at the extreme end of the age-at-onset distribution. Employing unbiased whole genome sequencing, we found *NOD2* and *LRRK2* variants that were shared by the twins. We then showed an epistatic interaction of the *NOD2* and *LRRK2* alleles identified in the twins and demonstrated that NOD2 signaling involves LRRK2. Combined, our results provide insight in the mechanism of leprosy susceptibility and highlight the contribution of specific amino acid alleles in *NOD2* and *LRRK2* in immune-mediated diseases.

## Results

### Whole genome sequence analysis

**Fig 1:**
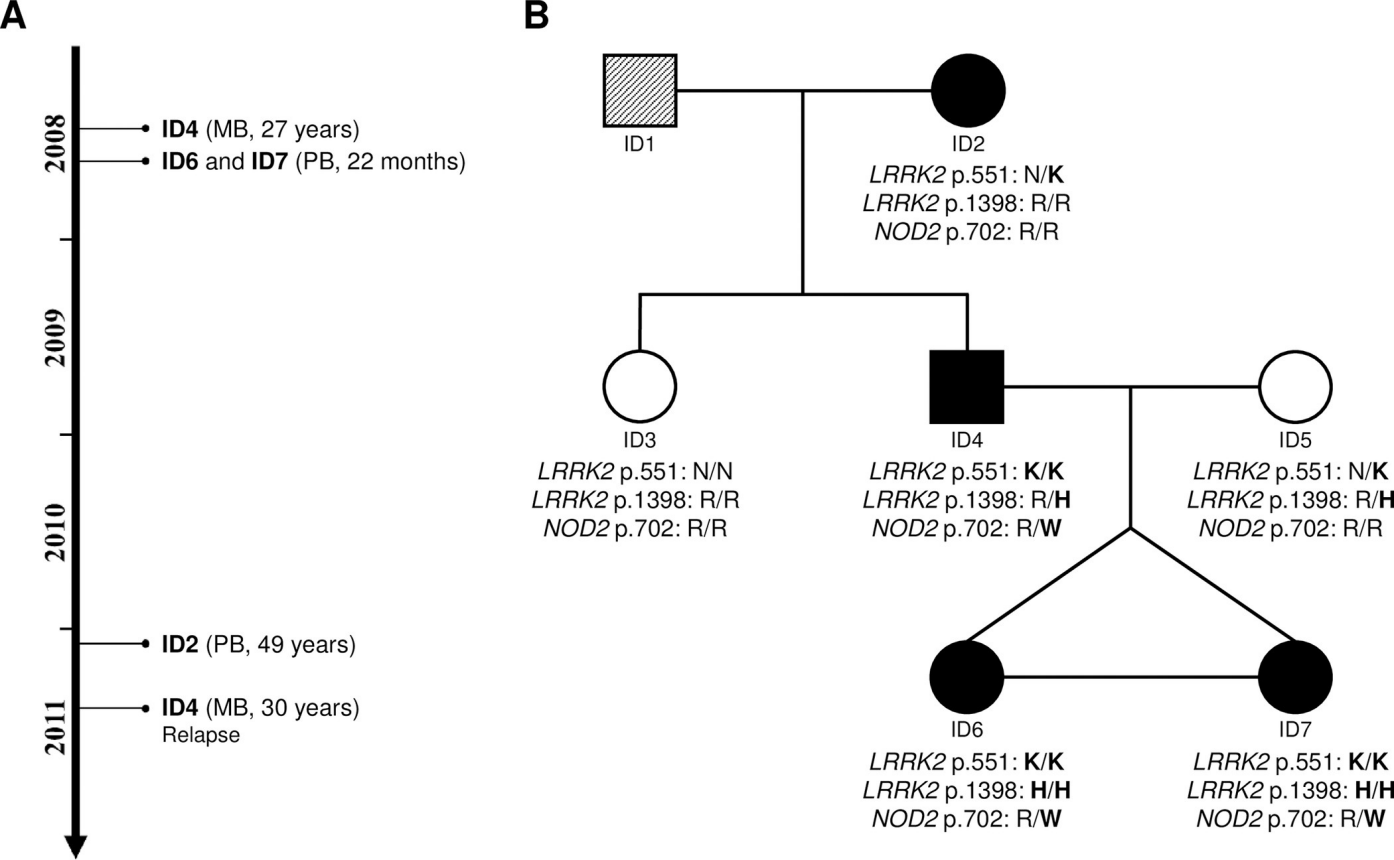
Missense variants in *LRRK2* and *NOD2* detected in a family with early onset leprosy in monozygotic twins. **(A)** Timeline of leprosy diagnosis among the affected family members, indicating the sample ID, leprosy subtype based on WHO classification and age-at-diagnosis. No patient developed leprosy reactions during treatment and a five-year follow-up. **(B)** Pedigree of the studied family indicating leprosy phenotype and genotypes of candidate variants *LRRK2* N551K (rs7308720), *LRRK2* R1398H (rs7133914), and *NOD2* R702W (rs2066844) in individuals with whole genome sequencing (WGS) data. Men and women are represented by boxes and circles, respectively. Leprosy patients (regardless of the clinical subtype) are indicated by filled symbols, while an unknown phenotype is indicated by a symbol with diagonal stripes. Monozygosity is represented by a horizontal line linking siblings. PB: Paucibacillary; MB: Multibacillary.

A small nuclear family from Northeast Brazil was identified with leprosy cases in three generations over a two-year period (Fig 1A). Unusually, monozygotic twins of age 22 months were both affected by leprosy with nearly identical distribution of skin lesions. The early age of onset for the twins together with the high prevalence of leprosy in the family suggested that host genetic factors may be involved in the familial clustering of the disease. Genomic DNA was obtained for six family members and used for whole genome sequencing (WGS; Fig 1B). The mean base coverage of all samples was 31±12 fold, with 91.5% of the genomes covered at least 10-fold (S1 Table). In total, nearly 8.4 million single nucleotide variants (SNVs) and short indels, including 44,679 coding and splice-site variants, were identified in the six family members. To systematically identify candidate leprosy susceptibility variants, we applied seven variant filtering strategies based on allele frequencies, mode of inheritance and allele distributions within affected family members according to age-at-diagnosis (S1 Fig). Next, variant-level and gene-level metrics were used to prioritize variants according to their *in-silico* predicted impact on protein function. In the final screening step, variants were prioritized according to the physiological function of their tagged proteins (see Materials and Methods).

Considering the low prevalence of leprosy and assuming recessive models of inheritance, we selected a minor allele frequency (MAF) of < 20% in any of the HapMap populations as cut-off (S1 Fig) [15,16]. We detected a total of 30 segregating non-synonymous and frameshift indels variants in 24 genes as leprosy risk candidates (S2 Table). Of these, five SNPs were predicted *in-silico* to encode protein-damaging variants: rs7271712 (T397M) in *SLC17A9*, rs7308720 (N551K) and rs7133914 (R1398H) in *LRRK2*, rs61740826 (C406Y) in *ZNF678* and rs2229531 (V200M) in *ACP5* (S2 Table). The twins and their father were homozygous for N551K in *LRRK2*; and only the early onset twins were homozygous for *LRRK2* R1398H, *SLC17A9* T397M, *ZNF678* C406Y and *ACP5* V200M. Among these variants, *LRRK2* N551K and R1398H had previously been shown to impact on LRRK2 protein activity and are established protective factors for CD and PD [7,17]. Conversely, the remaining three genes carrying homozygous missense variants in the twins–*SLC17A9*, *ZNF678* and *ACP5 –*had no known link to infectious diseases. Hence, the two *LRRK2* variants were considered the top candidates contributing to the early onset leprosy phenotype.

Under dominant models, 40 SNVs and Indels with MAF lower than 10% were identified as leprosy risk candidates, including eight variants prioritized as protein-damaging *in silico* (S1 Fig and S2 Table). Of the genes tagged by novel mutations only the *CR1* gene had prior evidence of a common variant impacting on human infectious disease risk, including leprosy [18,19]. The novel *CR1* E1674G amino acid change may deserve further attention in future studies. In addition, one variant (*NOD2* R702W, rs2066844) was a co-dominant risk factor for CD [20]. Variants near or within NOD2 have previously been associated with leprosy and T1R [7,21]. A follow-up of *NOD2* variants revealed three additional amino acid changes that had been excluded in the filtering approaches for segregation in the family: P268S, A612T and A725G (S2 Fig). The A725G variant was likely benign and there was no strong evidence for a risk effect of this amino acid mutations in common immune or infectious disease. In contrast, *NOD2* 612T, which was present in the affected father and grandmother but not in the twins, and P268S, which was considered to be too common to have a dominant effect on leprosy risk, had been associated with risk of CD [17,22].

We also searched for deletion structural variants (DSVs) that might contribute to leprosy susceptibility in the family (S1 Fig and S3 Table). In the DSV analysis, we identified 55 deletions with length ranging from 0.9 Kb to 43.4 Kb that overlapped coding regions of protein-coding genes. From this, four DSVs passed filtering for segregation in the family. DSVs overlapping *FSTL4*, *C18orf32* and *PARVB* were found in the three generations of the family where the affected individuals were heterozygous for the variants (S3 Table). Only the twins and their affected father carry the fourth candidate DSV, which overlapped with the *C9orf50* gene. There was no known involvement of these genes in mycobacterial or immune-mediated diseases.

Since both *LRRK2* and *NOD2* had been implicated in susceptibility to leprosy, CD and PD, and the corresponding proteins interact *in-vivo* in Paneth cells, we considered these two genes high priority candidates for exerting a joint effect on early onset leprosy susceptibility [10,23]. This choice did not exclude the possibility that additional susceptibility variants segregated in the family. *LRRK2* N551K and R1398H have global MAFs of 8.7% and 8.5%, respectively. However, their frequencies and linkage disequilibrium (LD) pattern vary among populations. In South Asians, both variants are of low frequency (MAF 4%) and present strong LD (*r*^*2*^ = 1), while in American and African populations they are more common (MAF between 14% and 16%), but with lower LD (*r*^*2*^ = 0.66 and 0.18, respectively) (S4 Table). *NOD2* R702W has global MAF of 2.6% and its MAF ranges from 0% in East Asian to 4.3% in European populations (S4 Table). Ancestry estimated based on principal component analysis of the family members of the present study and unrelated individuals from the five HapMap populations suggested a high African (AFR) ancestry for the grandmother (ID2) and shared American (AMR) and AFR ancestry for the remaining family members including the twins (S3 Fig). A reliable estimate of the genetic ancestry of family members is necessary to obtain the probability of encountering the *NOD2* and *LRRK2* genotypes observed in the family. Based on the allele frequencies in the AMR or AFR population and assuming complete LD between the two *LRRK2* variants, the probability of carrying the *LRRK2* and *NOD2* genotype combination of the twins (*LRRK2* N551K/R1398H homozygous + *NOD2* R702W heterozygous) was estimated at approximately 0.09%-0.10% in AMR and 0.03% in AFR individuals (S4 Table). Applying an approximate *r*^*2*^ = 0.5 between *LRRK2* N551K and R1398H for both population leads to an approximate estimate of 0.05% in AMR and 0.01% in AFR populations. Incidence of leprosy in the Northern Brazilian study population is 1.6x10^-4^ (Brazilian Health Ministry, Leprosy Epidemiological Record 2022). Countrywide, only 3% of all incident leprosy cases are children younger than 4 years. Combining genotype frequency with age-adjusted incidence suggests that the likelihood of finding additional early onset cases with the same genotypes can be estimated at approximately 1x10^-9^.

### LRRK2 variants affect ROS production and apoptosis in response to mycobacteria

**Fig 2:**
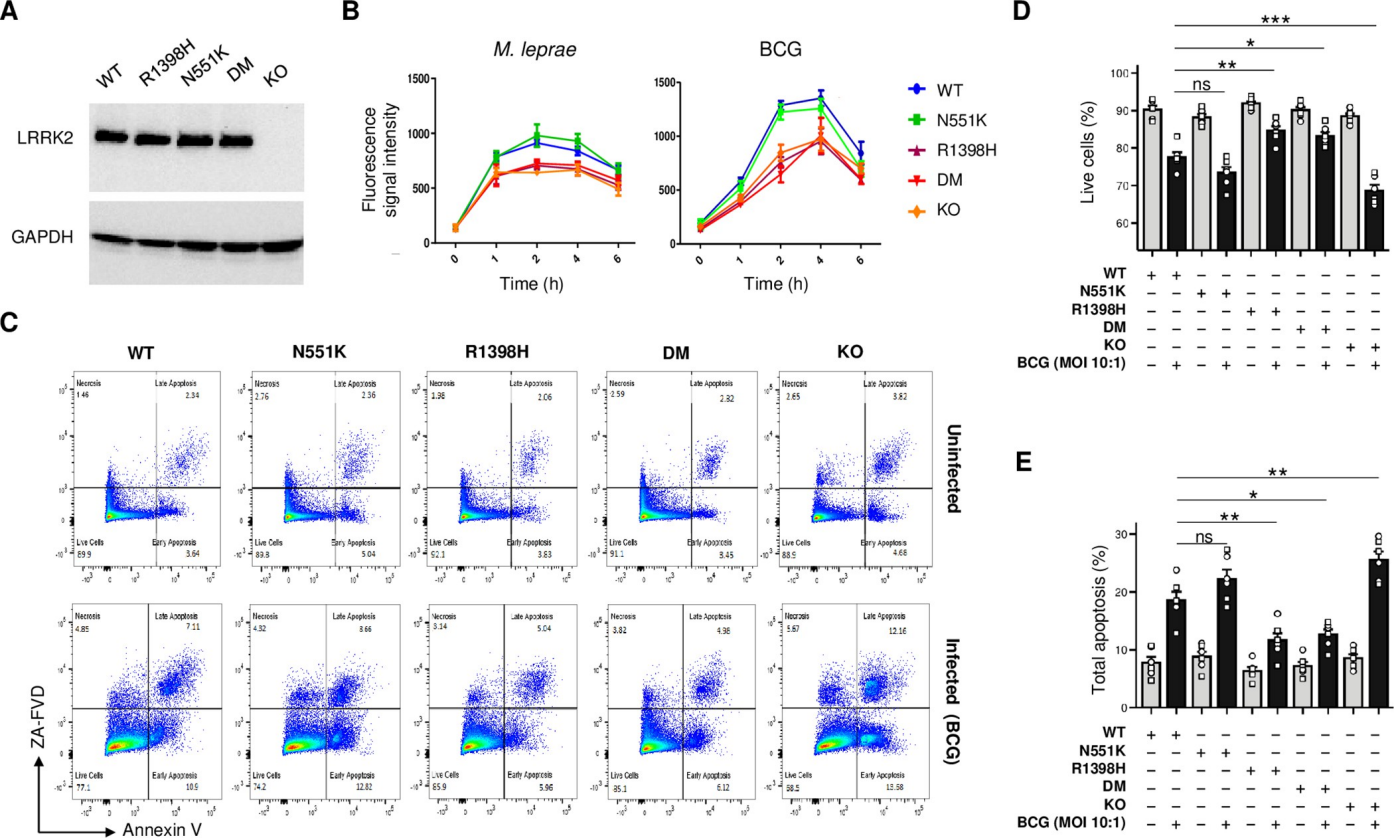
Effects of LRRK2 variants on respiratory burst and apoptosis in response to pathogens. **(A)** Protein levels in RAW264.7 cells of LRRK2 WT, LRRK2 mutants [N551K, R1398H and DM] and no LRRK2 protein (KO). The result is a representative of three independent experiments done by Western blot analysis. The expression of GAPDH is shown as a loading control. **(B)** Kinetics of reactive oxygen species (ROS) production in cells with different *Lrrk2* genotypes after stimulation with live *M*. *leprae* (left panel) or BCG (right panel). Results present the mean ± SE for three independent experiments (done in triplicates). **(C-E)** Effect of LRRK2 mutations on apoptosis in response to BCG compared to uninfected cells. **(C)** Flow cytometry for apoptosis, which is a representative of two independent experiments (done in triplicates) with similar results. **D-E** Percentages of live cells (**D**) and total apoptotic cells (**E**) were calculated in uninfected (grey bars) and BCG-infected cells (black bars) expressing different LRRK2 variants. (**D-E**) Data is presented as mean ± SE (n = 6). The two experiments are represented as circles and squares. ** 0.001 ≤ *P* < 0.01; * 0.01 ≤ *P* < 0.05.

To functionally validate the findings from the WGS analysis, we explored the impact of the N551K and R1398H LRRK2 variants on the cellular response to mycobacteria (Fig 2). The human and mouse LRRK2 proteins are highly conserved with approximately 90% amino acid sequence identity [24]. Hence, we applied CRISPR/Cas9 technology to knock-in the homozygous candidate LRRK2 variants in mouse RAW264.7 macrophages either individually or as double mutants (DM). A *Lrrk2* knock-out (KO) cell line was also generated as control. The expression of LRRK2 wild-type (WT) and corresponding mutant proteins in RAW264.7 cells is shown in Fig 2A. Oxidative burst, the generation of reactive oxygen intermediates (ROS), is an important mechanism by which intracellular mycobacterial growth is controlled [25]. Compared to LRRK2 WT, the LRRK2 R1398H mutation significantly reduced the production of ROS by RAW264.7 cells in response to live *M*. *leprae* infection (*P* < 0.001 at 1-2h post-infection [p.i.], *P* < 0.01 at 4-6h p.i.) while the N551K variant had no significant effect (Fig 2B). Interestingly, the LRRK2 DM had a similar effect on ROS production as the R1398H mutation (*P* < 0.001 at 1-2h p.i., *P* < 0.05 at 4h, p.i.), which suggested that the effect of the N551K/R1398H double mutation was mainly due to R1398H (Fig 2B). Consistent with a previous report, relative to LRRK2 WT expressing cells, the production of ROS was significantly lower in LRRK2 KO cells in response to infection (*P* < 0.01 at 1h p.i., *P* < 0.001 at 2-6h p.i., Fig 2B) [26,27]. We also investigated if mutant LRRK2 affected ROS production in response to Bacillus Calmette-Guérin (BCG) and we obtained similar results to those in response to *M*. *leprae* infection (Fig 2B). From these results, we concluded that BCG is a valid surrogate for *M*. *leprae* to study the effect of *LRRK2* and *NOD2* mutations on the host response to mycobacterial challenge.

Apoptosis is part of the innate immune response against mycobacteria [28]. Mutant LRRK2 variants or loss of LRRK2 expression have been linked to apoptotic cell death [29–31]. Hence, we tested if LRRK2 N551K, R1398H or DM affected BCG-induced apoptosis. As shown in Fig 2C–2E, in uninfected cells, mutant LRRK2 or absence of LRRK2 protein had no effect on naturally occurring apoptosis by RAW264.7 cells. However, following infection with BCG, apoptosis was significantly increased in LRRK2 KO cells compared to LRRK2 WT cells. In contrast, cells expressing LRRK2 R1398H displayed significantly reduced BCG-induced apoptosis compared to LRRK2 WT cells while LRRK2 N551K had no significant impact on the extent of BCG-induced apoptosis. Finally, expression of LRRK2 DM reduced BCG-induced apoptosis to a similar level as LRRK2 R1398H, suggesting that the effect of the double mutation on BCG-induced apoptosis is mainly due to the R1398H mutation (Fig 2C–2E). These results demonstrated how the same LRRK2 amino acid substitution can score as gain or loss of function variant depending on the read-out assay employed.

To investigate if LRRK2 heterozygous R1398H affects the cellular function, we generated a RAW264.7 cell line with heterozygous R1398H. We assessed the impact of homozygous 1398H/H (HOM) and heterozygous 1398R/H (HET) on apoptosis and ROS production in response to BCG challenge as compared to cells expressing WT LRRK2. In the absence of BCG stimulation, there was no difference across the genotype groups for ROS production (S4A Fig, 0h) and total apoptosis (S4B Fig, non-infected). Upon BCG challenge, we found strong reduction of ROS production (S4A Fig, 2-6h) and apoptosis (S4B Fig, BCG-infected) in HOM cells compared to WT cells. In direct comparison, HET and WT cells displayed no significant differences. However, a trend test across genotypes was significant suggesting a level of co-dominant control for the assay read-outs (S4 Fig).

### Effect of LRRK2 variants and NOD2 R702W on ROS production and apoptosis in response to BCG

**Fig 3:**
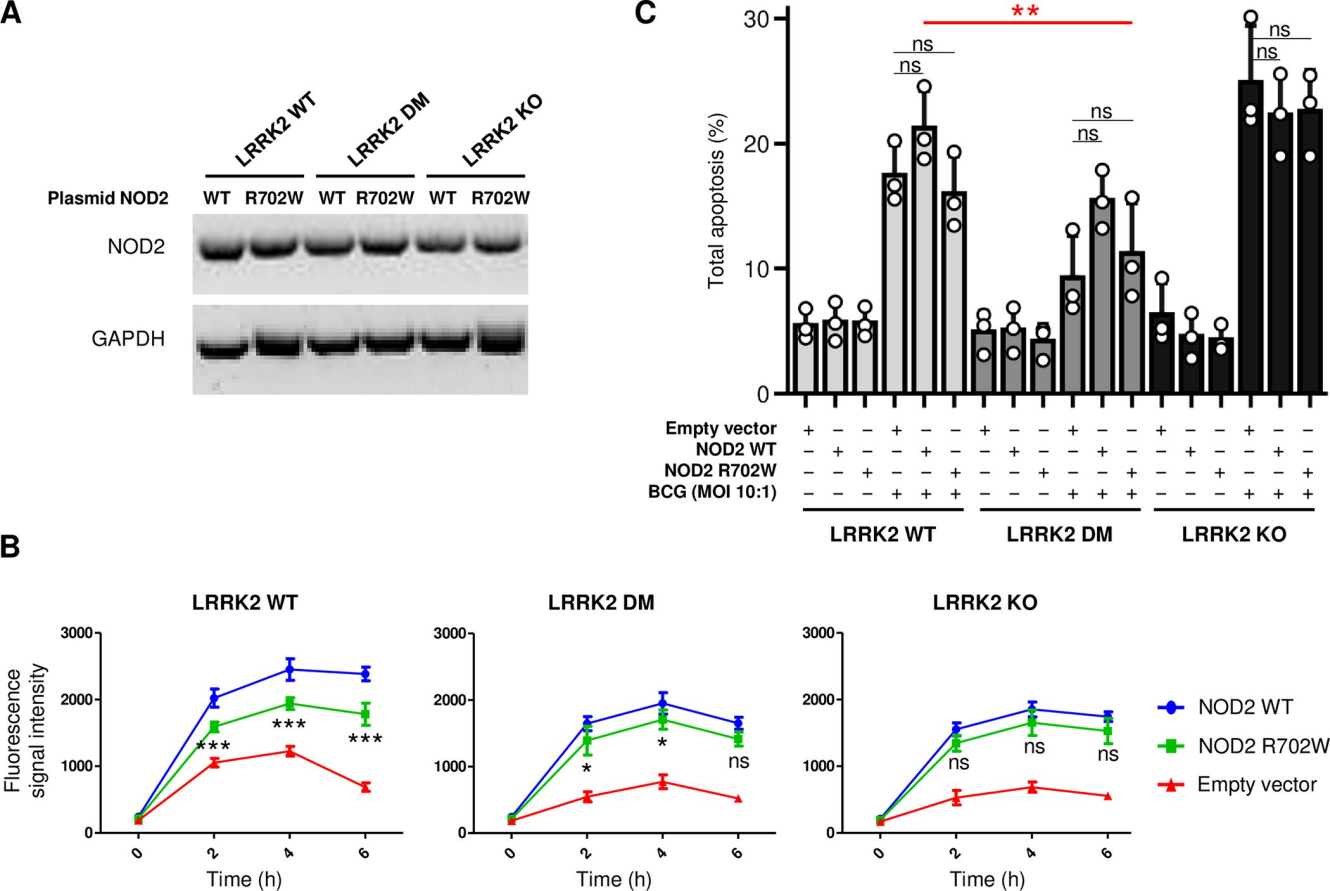
Effects of LRRK2 and NOD2 variants on respiratory burst and apoptosis in response to BCG infection. **(A)** RAW264.7 cells carrying LRRK2 WT, LRRK2 DM and no LRRK2 protein (KO) were transfected with plasmids expressing NOD2 WT or mutant NOD2 (R702W). One representative result of three independent experiments is shown. **(B)** Effect of NOD2 WT and R702W on reactive oxygen species (ROS) production after infection with BCG on the background of different LRRK2 genotypes. BCG-induced ROS production in cells transfected with NOD2 WT, NOD2 R702W or an empty plasmid are presented for cell lines with LRRK2 WT (Left panel), LRRK2 DM (middle panel) and LRRK2 KO (right panel). The graphs present one representative experiment (mean ± SD) of three independent experiments, each one done in triplicates. **(C)** Effect of LRRK2 and NOD2 variants on apoptosis in response to BCG. Percentage of total apoptotic cells, including cells with early and late apoptosis, was calculated in uninfected and BCG-infected cells, which is shown in the figure. The illustrated result presents one representative experiment (mean ±SD) of two independent experiments (done in triplicates). Significance of difference between LRRK2 WT+NOD2 WT and the genotype carried by the early onset leprosy twins (LRRK2 DM+NOD2 R702W) is indicated in red. **B-C** *** *P* < 0.001; ** 0.001 ≤ *P* < 0.01; * 0.01 ≤ *P* < 0.05; ns: non-significant. BCG: Bacillus Calmette–Guérin; DM: double-mutant; KO: knock-out; WT: Wild-type.

Since the twins and their father are heterozygotes for NOD2 R702W (Fig 1B), we investigated if NOD2 R702W and LRRK2 DM have a synergistic impact on ROS production and apoptosis. As mouse Nod2 does not carry an arginine (R) at position 702, it was not possible to introduce the human mutation into the mouse gene. To study LRRK2-NOD2 protein interactions, we opted to express the human NOD2 WT and mutant NOD2 proteins in the genome-edited RAW264.7 cells. Hence, plasmids expressing flag-tagged NOD2 WT, its variant R702W or an empty vector were introduced into RAW264.7 cells carrying LRRK2 WT, LRRK2 DM or not expressing LRRK2 protein (KO). No significant difference in NOD2 WT and NOD2 R702W protein expression was observed across cell lines (Fig 3A). Transfected cells were infected with BCG (MOI 10:1) and kinetics of ROS production were established. Overexpression of both NOD2 WT and NOD2 R702W proteins increased the ROS production in all three LRRK2 variant cell lines upon infection with BCG (Fig 3B). When overexpressed in LRRK2 WT cells, NOD2 R702W mediated significantly lower BCG-induced ROS production compared to NOD2 WT (*P* < 0.001 at 2-6h p.i.). When introduced in LRRK2 DM cells mutant NOD2 also mediated a lower induction of ROS (*P* < 0.05 at 2-4h p.i.). However, the R702W effect in LRRK2 WT cells was larger compared to LRRK2 DM cells (Fig 3B). When overexpressed in LRRK2 KO cells, NOD2 R702W did not significantly reduce ROS production relative to the NOD2 WT (Fig 3B). Taken together, our results showed that LRRK2 and NOD2 jointly modulated ROS production following BCG infection. However, biological significance of the effect will require additional study. Contrary to ROS production, overexpression of NOD2 WT or NOD2 R702W had no significant impact on apoptosis on any LRRK2 background (Fig 3C). A representative flow cytometry figure of the apoptosis experiments is presented in S5 Fig.

### LRRK2 variants and NOD2 R702W reduce NOD2-dependent RIP2 phosphorylation and NF-κB activity

**Fig 4:**
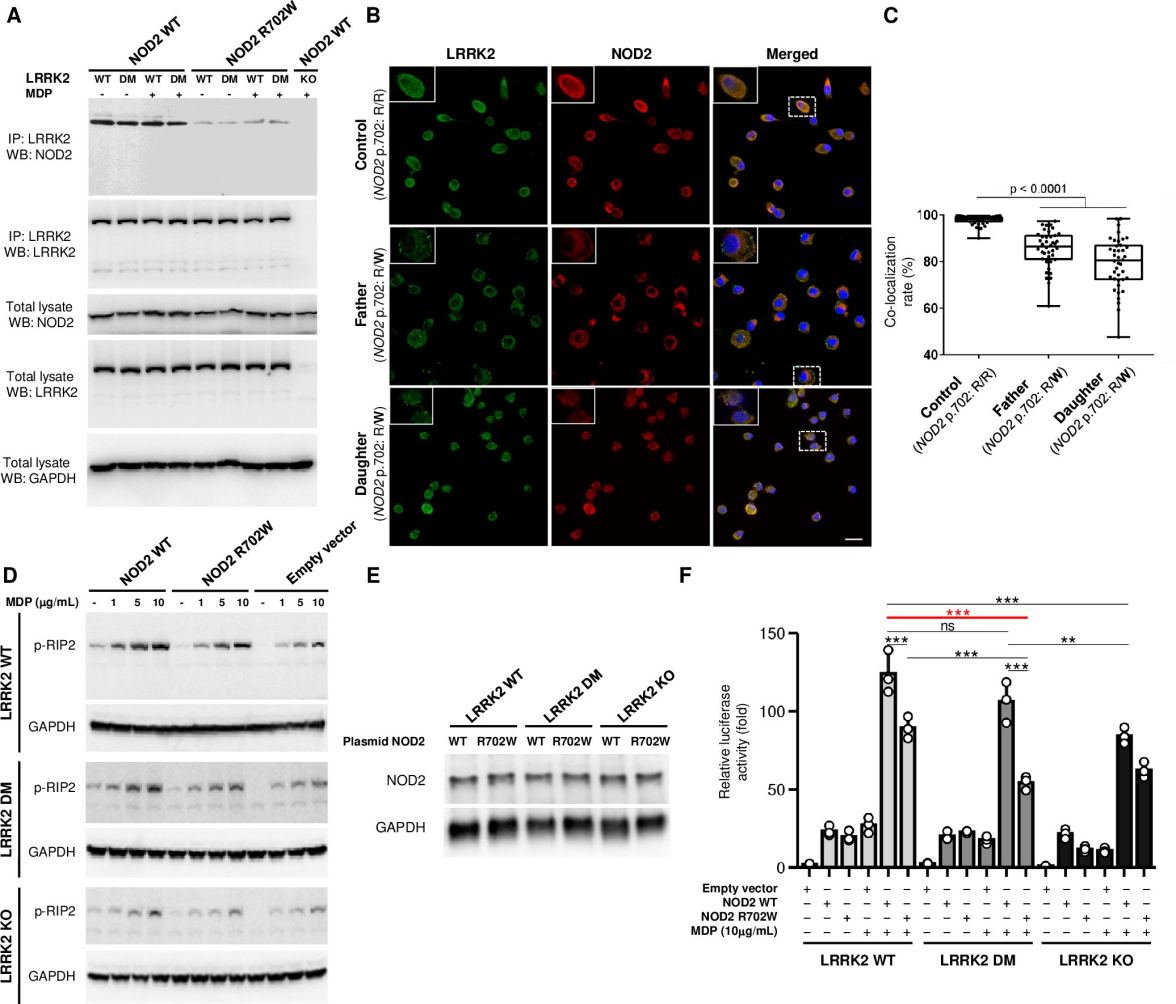
Effects of LRRK2 and NOD2 variants on LRRK2-NOD2 interaction, NOD2-dependent RIP2 phosphorylation and NF-κB activity. **(A)** Protein-protein interaction between endogenous LRRK2 and transfected NOD2 by Co-immunoprecipitation (Co-IP). Twenty-four hours post-transfection, cells were left untreated or treated with N-glycolyl MDP (10 μg/ml) for another 24 hours. Cell lysates were prepared and immunoprecipitated with a rabbit monoclonal antibody against LRRK2. Immunoprecipitants were analyzed by Western blot analysis with antibodies directed against LRRK2 and the FLAG tag of the fused FLAG-NOD2. The expression of LRRK2 and NOD2 using the same antibodies was also analyzed in the total lysate, where GAPDH expression was used as a loading control. LRRK2 KO RAW264.7 cell line transfected with a plasmid expressing NOD2 WT was used as a negative control. (**B-C)** Representative confocal images of LRRK2 and NOD2 co-localization in monocyte-derived macrophages. **(B)** Co-localization of LRRK2 (green) and NOD2 (red) was identified (yellow, merged) in macrophages of two family members (father and twin) with heterozygosity (R/W) for NOD2 p.702 and a control subject with homozygosity (R/R) for the common reference allele. Nuclei were stained with DAPI (blue). Scale bar represents 20μm in the figures and 10μm in the insets. (**C**) Quantification of total co-localization between LRRK2 and NOD2 was estimated from 40 cells by LAS X Software (Leica). Significance was calculated using one-way ANOVA, comparing each condition with the control using the Kruskal-Wallis test. Each dot represents a cell, the band in the box plot indicates the median, the box indicates the first and third quartiles and the whiskers indicate ± 1.5× interquartile range. (D) Effects of NOD2 mutation on MDP-induced phosphorylation of RIP2 at Ser 176 in cells with different LRRK2 genotypes. Twenty-four hours post-transfection, cells were left untreated or treated with different concentrations of MDP for another 24 hours. Phosphorylation of RIP2 (p-RIP2) was analyzed by immunoblotting with a specific antibody against phosphorylated at Ser 176 of RIP2. **(E)** NOD2 expression in the cells from panel D. The displayed results are representative of three independent experiments. The expression of GAPDH is shown as a loading control. (**F**) Effects of LRRK2 and NOD2 variants on NF-κB activity. The three RAW264.7 cell lines with different LRRK2 genotypes were transfected with *NOD2* plasmid (WT, R702W or empty vector) together with NF-κB firefly-Luc plasmid and Renilla-Luc plasmid pRL-TK (internal control). Twenty-four hours after electroporation, cells were left untreated or treated with 10 μg/ml of N-glycolyl MDP for another 24 hours. Cell lysates were subjected to luciferase assays. Results are expressed as relative luciferase activity (fold change), as compared with the luciferase activity of LRRK2 KO cells transfected with empty vector in the absence of N-glycolyl MDP. Results are presented as mean ± SD of a representative experiment (done in triplicate) of three independent experiments. Significance of difference between LRRK2 WT+NOD2 WT and the genotype carried by the early onset leprosy twins (LRRK2 DM+NOD2 R702W) is indicated in red. *** *P* < 0.001; ** 0.001 ≤ *P* < 0.01; * 0.01 ≤ *P* < 0.05; ns: non-significant.

We used co-immunoprecipitation (Co-IP) to determine if NOD2 interacts with LRRK2 in RAW264.7 macrophages and how the LRRK2 and NOD2 variants segregating in the study family affect this interaction. To test the impact of macrophage activation on the possible LRRK2/NOD2 interaction, we used the NOD2 ligand N-glycolyl muramyl dipeptide (MDP) as trigger [32]. Co-IP revealed that transfected NOD2 did interact with endogenous LRRK2, and this interaction was independent of the LRRK2 variant and MDP stimulation but sensitive to the NOD2 variant (Fig 4A). The interaction between LRRK2 and NOD2 proteins was confirmed by co-localization analysis with laser confocal microscopy (S6 Fig). Consistent with Co-IP, these co-localization results demonstrated that NOD2 R702W strongly diminished the interaction between LRRK2 and NOD2 in RAW264.7 macrophages (S6 Fig). To validate these results in human cells, we obtained PBMC from two family members and conducted a co-localization analysis of LRRK2 and NOD2 in monocyte-derived macrophages (MDMs) of the father and one of the twin girls as well as from a control subject (Fig 4B and 4C). MDMs were differentiated with GM-CSF and co-localization was analyzed after 24h of stimulation with N-glycolyl-MDP. In concordance to the results obtained with RAW cells, we observed a lower proportion of LRRK2-NOD2 co-localization in the macrophages from the father and daughter (NOD2 R702W heterozygote), compared to the control subject who was homozygous for the R702 reference allele (Fig 4B and 4C). Combined, our data showed impaired LRRK2-NOD2 interaction due to the NOD2 R702W variant in both murine and human macrophages.

NOD2 activation by MDP results in the activation of the obligate NOD2 kinase RIP2 (alias RIPK2) [33]. Upon interaction with NOD2, RIP2 becomes auto phosphorylated at two main sites, S176 and Y474. Phosphorylation of RIP2 results in the recruitment of TAK1 and subsequent activation of NF-κB [33]. We investigated the effect of the NOD2/LRRK2 interaction on RIP2 phosphorylation by Western blot using a RIP2 S176-specific antibody. RIP2 phosphorylation increased after transfection with NOD2 WT and NOD2 R702W constructs in an N-glycolyl MDP concentration dependent manner irrespective of the cellular LRRK2 background (Fig 4D). However, relative to NOD2 WT, RIP2 phosphorylation mediated by NOD2 R702W was substantially weaker irrespective of the LRRK2 variant carried by the cells even though no significant difference in the expression of NOD2 WT and R702W proteins was observed in these cells (Fig 4D and 4E). Compared to LRRK2 WT, LRRK2 DM reduced NOD2-driven phosphorylation of RIP2 (S7 Fig). Strikingly, LRRK2 KO resulted in decreased N-glycolyl MDP-induced RIP2 phosphorylation no matter if the cells were overexpressing NOD2 WT or NOD2 R702W (Figs 4D and S7). Combined, our results identified LRRK2 as important part of the NOD2 signaling cascade and demonstrated that the LRRK2 DM and NOD2 R702W mutations additively reduced RIP2 phosphorylation relative to wild-type proteins.

Next, we performed luciferase reporter assays to detect the combined impact of NOD2/LRRK2 variants on NF-κB activation. Consistent with the effect on RIP2 phosphorylation, N-glycolyl MDP-induced NF-κB activation increased in all three LRRK2 variant expressing cell lines overexpressing NOD2 WT or NOD2 R702W. In both LRRK2 WT and LRRK2 DM cells, NOD2 R702W triggered lower N-glycolyl MDP-induced NF-κB activation compared to NOD2 WT (Fig 4F). Cells devoid of LRRK2 or expressing LRRK2 DM displayed a strong trend of lower NF-κB activation relative to LRRK2 WT cells for both wild-type and mutant NOD2 (Fig 4F). Consequently, LRRK2 DM and NOD2 R702W had a cumulative effect on the reduction of N-glycolyl MDP-induced NF-κB activation and the variants carried by the early onset leprosy twins reduced NF-κB activation by approximately half compared to the wild-type variants expected in the general population (Fig 4F).

### Combined effects of LRRK2 DM and NOD2 R702W on cytokine production in response to BCG infection

**Fig 5:**
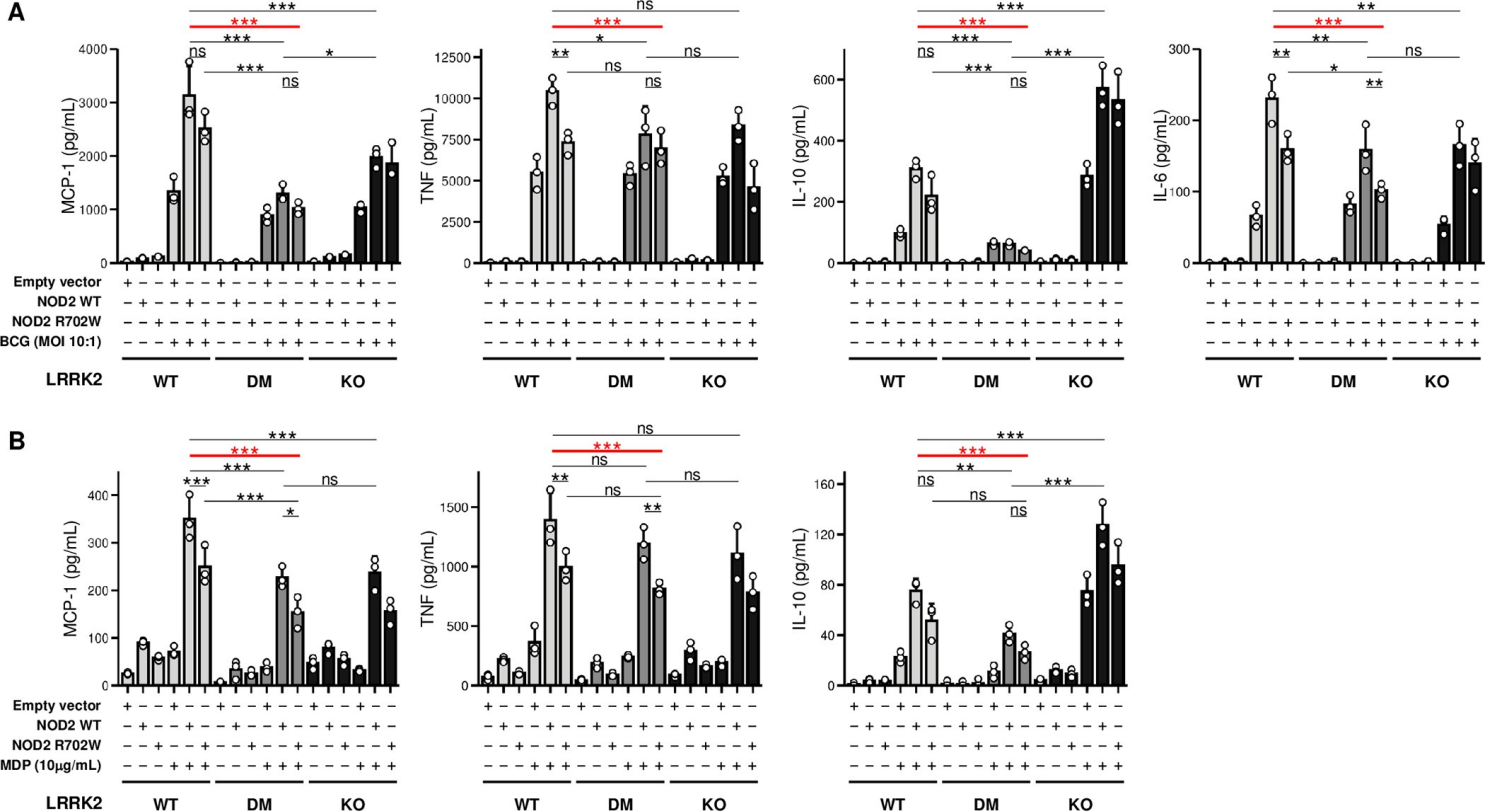
Effects of LRRK2 and NOD2 on cytokine secretion in response to BCG infection or stimulation with N-glycolyl MDP. Cell culture supernatant concentrations for MCP-1, TNF, IL-10 and IL-6 in cells expressing LRRK2 WT, LRRK2 DM (N551K+R1398H) or devoid of LRRK2 (KO) and transfected with NOD2 wild-type (WT), NOD2 R702W or empty vector. Secreted cytokines were measured following (**A**) infection with live bacillus Calmette–Guérin (BCG) or (**B**) stimulation with N-glycolyl MDP. The concentrations of IL-6 triggered by N-glycolyl MDP stimulation were below the detection limit and are not shown. **A-B** Results are presented as mean ± SD of a representative experiment done in triplicate of three independent experiments. Significance of difference between LRRK2 WT+NOD2 WT and the genotype carried by the early onset leprosy twins (LRRK2 DM+NOD2 R702W) is indicated in red. *** *P* < 0.001; ** 0.001 ≤ *P* < 0.01; * 0.01 ≤ *P* < 0.05; ns: non-significant.

Next, we asked to what extent LRRK2 DM and NOD2 R702W modulate cytokine/chemokine production. The release of four key mediators (MCP-1, TNF, IL-10, and IL-6) into the supernatant of cell cultures was measured before and after infection with BCG or stimulation with MDP. As expected, infection with BCG induced a stronger cytokine/chemokine response than stimulation with N-glycolyl MDP only (Fig 5). Moreover, the additive effect of NOD2 (WT or R702W variant) overexpression was more pronounced for MDP stimulation. In the absence of transfected NOD2, BCG infection triggered secretion of MCP-1 and TNF while stimulation with N-glycolyl MDP did not, which is consistent with its role as NOD2 ligand. N-glycolyl MDP-triggered secretion of IL-6 remained below the limit of detectability. Release of MCP-1, TNF and IL-6 was substantially reduced by both LRRK2 DM and NOD2 R702W relative to wild-type proteins. There was no significant difference between LRRK2 DM and LRRK2 KO cells, except for the BCG-triggered secretion of MCP-1, which was lower for LRRK2 DM compared to LRRK2 KO (Fig 5A).

An unexpected observation was the impact of LRRK2 on the release of the anti-inflammatory cytokine IL-10. RAW264.7 cells expressing LRRK2 DM displayed strongly reduced secretion of IL-10, which was not significantly increased following NOD2 overexpression. Strikingly, absence of LRRK2 resulted in an increased release of IL-10 for both BCG and N-glycolyl MDP stimulation relative to either wild-type or mutant LRRK2-expressing cells even in the absence of overexpressed NOD2 (Fig 5). This observation together with the NOD2-independent impact of LRRK2 mutations on apoptosis activity suggested a pronounced immune regulatory role of LRRK2 (Fig 2). Collectively, our data show that NOD2 signaling is dependent on the interaction with functional LRRK2. While these observations deserve additional study, the important observation in the context of the present paper was the additive effect of both LRRK2 and NOD2 variants shared by the twins on the release of major mediators of the immune response.

## Discussion

By applying unbiased WGS analysis on a family with monozygotic twins with extreme early onset leprosy, we identified three coding variants in the *LRRK2* and *NOD2* genes as strong candidates for contributing to early onset leprosy susceptibility. By identifying specific amino acid changes as risk factors for leprosy, these results substantially expand our understanding of the role of these multifunctional proteins in disease pathogenesis. While we have investigated only a single family, a strong case can be made that a single patient with a genetic lesion that is properly followed by functional validation can provide useful insights into mechanisms of disease susceptibility [34]. Common SNPs in the genomic vicinity of *NOD2* have been associated with leprosy in multiple studies [7,21,35–39]. None of these studies provided a functional validation of these associated common variants. Given the presence of additional strong candidates in the *NOD2* vicinity (e.g. *SNX20*) and the notorious difficulties in identifying the gene target of common SNPs (LD, long range enhancer effects), our results are the first to implicate directly *NOD2* in leprosy susceptibility. Similarly, the *LRRK2* gene has mainly been implicated in leprosy susceptibility by association studies without any functional follow-up [7,31,40–44]. However, the *LRRK2* M2397T mutation is connected to altered biological function of *LRRK2* and associated with leprosy and excessive inflammatory responses in leprosy [41,42]. The latter associations may have been confounded by LD with the *LRRK2* R1628P mutation which is also affecting *LRRK2* function and strongly associated with both of the former leprosy phenotypes [31,43,44]. The R1628P mutation is found only in East Asian populations (at low frequency) and therefore cannot play a role in other populations. Our present results provide the first evidence for a role of *LRRK2* in leprosy risk outside of East Asia by invoking trans-ethnicity *LRRK2* amino acid mutations. Intriguingly, the same *LRRK2* coding variants identified in the leprosy family have been implicated in reduced susceptibility to PD and CD while *NOD2* R702W is a risk factor for both CD and PD [17]. Hence, our findings expanded the overlap in the genetic control of these three diseases to specific amino acid substitutions and emphasized intersecting mechanisms of pathogenesis [9]. By employing a functional follow-up of the genetic findings, our study demonstrated the individual and joint effects of *LRRK2* and *NOD2* on the innate immune response. Of the two *LRRK2* mutations, R1398H, the only *LRRK2* variant homozygous only in the early onset twins, displayed a predominant functional impact. This was consistent with previous results linking R1398H with increased GTPase activity and Wnt signaling and strengthened the candidacy of R1398H as leprosy susceptibility factor [17,45]. While our study was motivated by early onset leprosy, it is likely that the additive interactions of the LRRK2 and NOD2 variants and their effect on key inflammatory host pathways are also valid in PD and CD patients.

*LRRK2* and *NOD2* genes are highly expressed in immune cells in different tissues including the gut, blood and brain [46]. Here, we decided to probe the impact of the LRRK2 and NOD2 variants on three aspects of anti-mycobacterial host immunity: ROS production, apoptosis, and secretion of immune-modulatory chemokines/cytokines. In addition to being a key effector mechanism in anti-mycobacterial host responses, production of ROS and the resulting oxidative stress are also key events in CD and PD pathogenesis [47–49]. Apoptosis plays a vital role in host defense against intracellular pathogens, including mycobacteria [50,51]. Apoptosis is present in leprosy lesions and may contribute to nerve damage [52,53]. Similarly, apoptosis is a key event of IBD pathogenesis and neuronal cell death in PD [54,55]. Cytokine responses to *M*. *leprae* determine the clinical manifestation of leprosy and dysregulation of cytokine-mediated inflammatory host responses has been implicated in PD and CD susceptibility [56–59]. Importantly, both LRRK2 and NOD2 had previously been implicated in these three investigated host response pathways. LRRK2 modulates apoptotic activity of macrophages following infection with BCG [31]. Both LRRK2 and NOD2 are modulators of ROS production by immune cells following pathogen challenge [17,31,60,61]. The loss of pro-inflammatory activity of LRRK2 and NOD2 has been linked to leprosy, PD, and CD, and *LRRK2* enhances NOD2-mediated inflammatory cytokine production [17,62,63]. In the present study, we identified specific variants of LRRK2 and NOD2 that contributed to the functional effects on innate host immunity.

While NOD2 signaling and ROS production were significantly modulated by the LRRK2—NOD2 interaction, apoptosis was not. LRRK2 is an inhibitor of apoptosis and IL-10 secretion, and these were the only readouts where the R1398H mutation presented as gain-of-function. Conversely, in assays that showed an additive effect of NOD2 and LRRK2, i.e. ROS production, N-glycolyl MDP-triggered induction of NF-κB activity as well as N-glycolyl MDP and BCG-triggered pro-inflammatory chemokine and cytokine production, the LRRK2 variants were loss-of-function mutations. While the interaction of LRRK2 and NOD2 was more strongly dependent on the NOD2 R702W mutation compared to the LRRK2 variants, it seemed likely that even in the presence of intact physical interaction, the functional integrity of the complex was reduced. This conclusion was consistent with the impact of the LRRK2 DM on NOD2 signaling, the induction of NF-κB activity and cytokine secretion. Neither the N551K nor the R1398H substitution reduce LRRK2 kinase activity [17]. Conversely, we had previously shown that the gain-of-kinase activity LRRK2 1618P mutation is a gain-of-function variant for both ROS production and apoptosis [31]. Taken together these results suggested that LRRK2-mediated ROS production may be dependent on LRRK2 kinase activity while increased apoptosis inhibition is not.

We showed that LRRK2 is an important part of the NOD2 signaling cascade in RAW cells. While the use of a murine cell line is a potential limitation of our results, mice have been widely used to study LRRK2 and NOD2 function. Of note, the observation that LRRK2 and NOD2 interact in an allele-specific fashion has been validated in primary human cells in our study. Results obtained with RAW cells are also consistent with the observation that microglia from *Lrrk2* KO mice displayed a reduced inflammatory response after treatment with α-synuclein pre-formed fibril or LPS [64]. In a mouse model of colitis induced by dextran sodium sulfate, LRRK2 lies downstream of the β-glucan receptor Dectin-1 and overexpression of LRRK2 leads to the activation of the NF-κB components, TAK1 complex and TRAF6, and the enhancement of pro-inflammatory cytokine secretion [65]. Co-immunoprecipitation assays revealed an interaction between LRRK2 and NOD2 when both proteins were overexpressed in HEK293T cells. This interaction also occurred between the endogenous LRRK2 and NOD2 in Paneth cells to properly secret antimicrobial peptides into the intestinal lumen and promote gut-microbiota homeostasis [23]. Moreover, LRRK2 enhanced the phosphorylation of RIP2 at Ser 176 and promoted NF-κB activation, augmenting the production of pro-inflammatory cytokines upon NOD2 activation [63]. We confirmed and expanded on these previous observations and showed that the LRRK2 and NOD2 mutations synergistically reduced RIP2 phosphorylation and NF-κB activation. Finally, while Ser 176 phosphorylation is essential for RIP2 kinase activity, our results obtained with LRRK2 DM and LRRK2 KO cells confirmed that NF-κB activation may also occur at reduced levels independently of RIP2 phosphorylation [66,67].

As expected from the RIP2 phosphorylation and NF-κB activation experiments, we found that the LRRK2 and NOD2 mutations significantly impaired MCP-1, TNF, IL-6 and IL-10 secretion by RAW cells upon stimulation with BCG or N-glycolyl MDP. The chemokine MCP-1 is involved in recruiting macrophages and monocytes to the sites of infection, and thereby enhances innate inflammatory events. In addition, MCP-1 exerts pleiotropic functions on immune cells, including stimulation of cellular differentiation, proliferation and survival, as well as activating phagocytosis, and efferocytosis [68]. The presence of elevated serum levels of MCP-1 in lepromatous leprosy patients and the increased expression of MCP-1 in the skin lesions of leprosy patients suggest this chemokine as important player in the pathogenesis of leprosy [69,70]. Kipnis *et al*. demonstrated that low-dose aerosol *M*. *tuberculosis* infection of mice deficient in MCP-1 resulted in entry of macrophages in the lung and a transient increase in bacterial load [71]. Taken together, the data suggested MCP-1 as a protective factor against leprosy.

TNF is a key pro-inflammatory cytokine in mycobacterial infections that triggers granuloma formation while inhibiting mycobacterial growth [72]. In humans, primates and mice, TNF plays a critical role in containment of chronic and latent *M*. *tuberculosis* infection [73]. Accordingly, anti-TNF immunotherapy, which is used for the treatment of autoimmune and chronic inflammatory diseases, disrupts effective immunity against *M*. *tuberculosis* and therefore increases the risk of latent TB reactivation [74,75]. It has also been reported that anti-TNF therapy is associated with development of lepromatous leprosy and T1R [76,77]. *TNFA* genetic variants are classic risk factors for leprosy and its gene product, TNF, is a major signature cytokine for the tuberculoid pole [78,79]. TNF is elevated in the blood of both PB and MB leprosy, and is observed in granulomatous lesions [80,81]. Elevated circulating TNF has also been observed in patients with leprosy reactions as compared to patients without reactions, suggesting a role of TNF in acute inflammatory episodes in leprosy patients [82].

IL-6 plays important role in inflammation and activation of Th1 and Th17 cells, which are involved in controlling *M*. *leprae* infection and thereby influence the clinical manifestations of leprosy [83,84]. While increased expression of IL-6 is implicated with excessive pro-inflammatory episodes such as T1R, deficiency of IL-6 led to increased bacterial loads in an animal model of mycobacterial infection [38,85–88]. In humans, anti-IL-6 treatment increased the risk of mycobacterial infection [89]. Paradoxically, IL-6 is also known to promote the intracellular growth of *M*. *avium* and IL-6 produced by *M*. *tuberculosis*-infected macrophages inhibited responsiveness of uninfected macrophages to IFN-γ [90,91]. Hence, it is possible that IL-6 modulates the host immune response depending on the nature of the challenge.

IL-10 is an anti-inflammatory cytokine that plays a key role in infections by preventing inflammatory damage to host tissues [92–95]. Up-regulation of IL-10 expression by IL-27 suppresses IFN-γ-induced antimicrobial activity against *M*. *leprae* [96]. IL-10 secreted by different immune cells plays important roles in the progression and phenotype of leprosy through regulation of both innate and adaptive immune responses [97–99]. In addition, variant −819 C/T (rs1800871) in the promoter region of *IL10* has repeatedly been associated with susceptibility to leprosy [100,101]. All these data suggested an important role of IL-10 in regulation of immune response and the progression of leprosy. Finally, in our experiments LRRK2 DM as well as LRRK2 KO had a detrimental impact on secretion of pro-inflammatory cytokines by RAW macrophages. However, a recent study of long-term *M*. *tuberculosis*-infected LRRK2 KO mice observed increased transcription of pro-inflammatory cytokines in the lungs of these animals [102]. These divergent results suggested, as previously pointed out by Shutinoski *et al*, that the outcome of the LRRK2-pathogen interaction may depend on both the pathogen and the length of interaction [103].

Our results demonstrate how variants in *LRRK2* and *NOD2* can have a joint effect on different aspects of the immune response which may lead to early onset leprosy. Nevertheless, we cannot exclude the possibility that the remaining candidate variants detected in our study are involved in leprosy susceptibility. For example, the novel variant (E1674G) in *CR1*, a gene recently associated with leprosy, is a possible candidate for involvement in leprosy that deserves further follow-up [19]. The remaining variants are located in genes of unknown function or with no clear involvement in leprosy or mycobacterial infections. Yet, we cannot exclude their possible role in leprosy susceptibility. While we cannot exclude the presence of additional risk factors, the functional validation of *LRRK2* and *NOD2* variants implicates these amino acid changes in early onset leprosy. We consider the presence of more than three risk factors in a single family unlikely, which does support an exclusive role of the studied variants in early onset leprosy in this family.

In previous work, it was shown that the LRRK2 1628P mutation was a risk factor for leprosy but protective for T1R [31,44]. The antagonistic pleiotropic effect of the LRRK2 1628P mutation is a reflection of the two sides of the anti-pathogen host response. Initially a beneficial host response is directed against the infecting pathogen. However, an excessive host response will lead to host cell damage and this host damaging response can occur after the elimination of the pathogen as is the case for leprosy and T1R and other infectious diseases [104]. Our present data extended the concept of antagonistic pleiotropy of LRRK2 mutations to leprosy and PD/CD. The two mutations were a loss-of-function for BCG and N-glycolyl MDP-triggered cytokine production and also linked to a reduced respiratory burst response and reduced apoptosis. Since leprosy is an infectious disease, a weakened inflammatory host response mediated by the LRRK2 DM and NOD2 R702W is expected to increase susceptibility. Conversely, both PD and CD are inflammatory disorders corresponding to T1R. Given that NOD2 is a microbial sensor and the *LRRK2* and *NOD2* mutation mediated a dampened classical anti-microbial host response this suggested the involvement of microbes in early events of both CD and PD. The latter conclusion is consistent with recent observations in animal models of PD [103,105]. Indeed, it is plausible that antagonistic pleiotropy across different diseases does contribute to the maintenance of genetic risk factors in human populations. The results of our study highlight the need for a better understanding of pleiotropy and possible epistatic effects in the dissection of the pathogenesis of common inflammatory disorders.

## Materials and methods

### Ethics statement

All participants, or their legal representative, provided written informed consent independently and agreed to donate specimens for this study. This study was approved by Research Ethics boards of the Pontifícia Universidade Católica do Paraná (CEP 169.382 and 1.709.543) and the Federal University of Piauí (CEP 657.779).

### Study participants

A three generational family comprising seven family members–including four leprosy cases–was enrolled from Piauí state, Northeast Brazil. The index case was the father who was diagnosed with multibacillary (MB) leprosy in 2008 at the age of 27 years and a second time in 2011. Following the first leprosy diagnosis of the father, household contact tracing was conducted. As part of this follow-up, both twin girls were diagnosed with paucibacillary (PB) leprosy at the age of 22 months by two independent experienced leprologists. In 2011, the grandmother was diagnosed with PB leprosy at the age of 49 years. All individuals in the family had been BCG vaccinated. No patient developed leprosy reactions during treatment and a five-year follow-up. No additional mycobacterial diseases were detected in any family member. Both the paternal aunt (ID3) and the mother (ID5) of the twins remained unaffected. However, only the mother had been in prolonged contact with the three leprosy cases. Therefore, only the mother was included in the variant filtering approaches as unaffected control.

### Whole genome sequencing

WGS was performed for six family members (ID2 to ID7 from Fig 1B) on HiSeq 2500 platform (Illumina) to generate paired-end 150 bp reads at the Genome Quebec and McGill Innovation Centre. Quality assessment of the raw data was performed using FastQC v0.11.4 software (Babraham Bioinformatics; http://www.bioinformatics.babraham.ac.uk/projects/fastqc/). The reads were mapped to human genome reference GRCh37+decoy using the BWA-mem algorithm on BWA v0.7.12 [106]. Mapped reads were sorted according to their genomic coordinate position and PCR duplicates were flagged using Picard v1.134 (Broad Institute, GitHub Repository; https://github.com/broadinstitute/picard). Next, local realignment around indels and base recalibrations were performed using GATK v3.5 [107]. Quality assessment of the mapped reads was performed using QualiMap v2.1.1 [108]. GATK HaplotypeCaller was used to call SNVs and short indels for each sample, followed by GenotypeGVCFs for the six samples together. Variant Quality Score Recalibration from GATK, using default parameters was used to reduce the amount of false positive [109]. Genotypes with genotype quality (GQ) lower than 20 were removed. Finally, the variants were annotated using wANNOVAR (2016) [110].

Deletion structural variants (DSVs) in autosomal chromosomes were detected using Genome STRiP v2.0 SV pipeline with default parameters [111]. For that, a total of 25 high-coverage WGS samples from the 1000G database were included in this step to run together with the 6 samples from the studied family [112]. Next, GATK was used to apply hard filtering to remove low quality deletions as follow: GSELENGTH < 200; GSCLUSTERSEP ≤ 2.0 or NA; GSM1 ≤ 0.5 or ≥ 2.0 or NA; GLINBREEDINGCOEFF < -0.2; GSNONVARSCORE ≥ 13.0; GSDUPLICATESCORE ≥ 0 or DOSAGE_CORRELATION ≥ 0.5; call rate ≥ 80%. Overlap of the DSVs to protein-coding genes was annotated using GeneOverlap command on Genome STRiP.

To identify the population structure of the studied family, principal component analysis (PCA) was performed using PLINK v1.9 [113]. For that, genotypes from VCF files were converted to PLINK format using BCFtools (http://github.com/samtools/bcftools). Autosomal variants were pruned based on LD (window size of 50kb, step size of 5 and variance inflation factor of 1.5) and MAF > 10% as implemented in PLINK. Variant pruning was done using the samples from the 1000 Genomes Consortium (1000G) representing the five super populations worldwide: African/African American, Admixed American/Latin, East Asian, South Asian and European [15]. From the 428,824 pruned variants, A/T and C/G SNPs and variants absent from the studied family were excluded. In total, 237,150 variants were used for PCA analysis including 2,504 unrelated individuals from 1000G and the six family members from the present study.

### Candidate variant detection from WGS data

Five filtering steps (A to E) were used to select variants based on A) the variant location within protein-coding genes (coding or splice-site variants), B) its type (missense, nonsense, frameshift indels or splice-site variant), C) inheritance model (dominant or recessive), D) variant frequency in population samples from the database, and E) age-at-diagnosis of the affected family member. To apply filtering step D, we searched the variant frequency in the following population samples from the 1000G and Exome Aggregation consortium (ExAC) databases: African/African American, Admixed American/Latin, East Asian, South Asian and European (Non-Finnish European from ExAC) [15,16]. Using different parameters/thresholds in steps C to D, we implemented seven custom filtering approaches to identify candidate variants (S1 Fig). Filtering approaches #1 to #4 were designed to detect variants in the recessive model, while filtering approaches #5 to #7 to detect variants in the dominant model (S1 Fig). Different thresholds of variant frequencies were used in the four approaches following the recessive model, while in the dominant model, we selected variants that were not reported in the 1000G nor ExAC databases (See S1 Fig). Based on the age-at-diagnosis of the leprosy patients in the family (Fig 1A), in filtering step E we searched for variants present in i) all the affected family members, regardless of the age-at-diagnosis (Filtering approaches #1 and #5 in S1 Fig); ii) the father and the twins, which are the cases younger than 30 years (Filtering approaches #2 and #6 in S1 Fig) and iii) only in the twin girls, that are the early-onset cases in the family (Filtering approaches #3, #4 and #7 in S1 Fig). An additional search for candidate variants segregating in the family was done employing known leprosy genes [10]. We included in this analysis a total of 86 genes previously associated with leprosy in GWAS and/or target association studies up to January 2023 (S5 Table). Filtering steps were applied to identify candidate variants in these genes as implemented in the WGS data, but with a less stringent threshold for variant frequency in databases in step D (S1 Fig). For known leprosy genes, thresholds of MAF < 20% and MAF < 10% in the four populations from 1000G/ExAc were used in the recessive and dominant models, respectively (S1 Fig). Genotype validation of candidate variants was done as described in the S1 Method.

Once candidate variants were identified, variant-level and gene-level metrics based on computational prediction were used to prioritize the variants that are most likely to have an impact on the protein structure and function. For that, we used PolyPhen-2 v2.2.2r398 and CADD v 1.4 as variant-level metrics, and GDI (2016) as gene-level metric [114–116]. We focused on variants that presented the three following criteria: i) it has scaled CADD score ≥ 20 (Top 1% most deleterious), ii) it is a missense variant with PolyPhen-2 HumVar score > 0.446 (possibly or probably damaging), a nonsense variant, frameshift indel or splice-site variant and iii) it is located in a gene with GDI score < 13.84 (medium or low GDI). Among compound heterozygous variants (Filtering approach #4 in S1 Fig), we prioritized genes with GDI score < 13.84 where both variants reached criteria one and two. Linkage disequilibrium (LD) estimates between *LRRK2* N551K and R1398H were performed using Haploview software v4.2 based on genotyping data from the five populations from 1000G [15,117]. Estimated frequencies of LRRK2 R1398H and NOD2 R702W combined genotypes were obtained assuming Hardy-Weinberg equilibrium for both SNPs and using MAF from gnomAD database [118].

For detection of candidate DSVs, deletions reported as overlapping with exon, CDS or gene were selected. Then, we applied the same filtering approaches as used for SNV and short indels for the recessive and dominant model (S1 Fig). DSV with call-rate < 80% in the 31 samples were excluded. We searched whether the candidate deletions were present on Database of Genomic Variants (DGV) catalog and kept those that were found in less than 50% of the sequenced samples from 1000 Genomes database (S1 Fig) [15,119].

### Genome-editing with CRISPR/Cas9

1. Synthesis of gRNAs: The gRNAs for generation of LRRK2 N551K, R1398H were synthesized by using GeneArt precision gRNA synthesis kit (Thermo Fisher) according to the manufacturer’s instruction. Prior to making gRNAs, 34-nucleotide forward and reverse target DNA oligonucleotides were designed using the CRISPR search and design tool (Thermo fisher) and synthesized (S5 Table). Then the gRNA DNA templates were PCR assembled and gRNAs were synthesized by *in vitro* transcription. The gRNAs were purified and their concentrations were measured. TrueGuide Synthetic sgRNA for generation of *Lrrk2* KO cell line was purchased from Thermo Fisher (Assay ID: CRISPR206078_SGM).

2. Electroporation: One day prior to transfection, RAW264.7 cells were split into a new flask with fresh growth medium such that the cells reach 70–90% confluent the following day. On the day of electroporation, cells were washed with PBS (without Ca^2+^ and Mg^2+^), digested with 0.25% trypsin-EDTA for 8–10 min at 37°C. After neutralization with growth medium, the cells were counted and appropriate amounts of cells (1 x 10^5^ cells per transfection) were transferred to a 1.5 ml microcentrifuge tube. The cells were washed once with PBS by centrifugation at 500g for 5 min. At the same time as preparation of cells for electroporation, 2μg Cas9 protein and 400 ng gRNA were mixed in 10 μl of resuspension buffer R and incubated at room temperature for 10 min. Prepared cells were re-suspended in the buffer R containing Cas9-gRNA complex and 50 pmol of donor HDR templates (S6 Table) was added. Cell mixture was transferred into a 10 μl Neon tip with Neon pipette and electroporation were performed using the parameters as following: pulse voltage 1680 V, pulse width 20 ms and pulse number 1. After electroporation, cells from two Neon tips were immediately mixed into prewarmed 1 ml growth medium in a well of 12-well plate and cultured for 4 days.

3. Restriction fragment length polymorphism (RFLP) assay: Genomic DNA was extracted from RAW264.7 cells transfected with Cas9-gRNA and donor HDR templates. Genomic DNA was then PCR amplified with primers flanking the donor target region (see PCR primer sequences in S6 Table). The amplification was carried out with AmpliTaq Gold 360 master mix (Thermo Fisher), using the following cycling condition: 95°C for 10 min for initial denaturation; 40 cycles of 95°C for 30s, 60°C for 30s and 72°C for 35s; and a final extension at 72°C for 7 min. Then, 1 μg PCR products were digested with 10 U of BstUI at 60°C or AvaI at 37°C overnight and resolved on 1.2% agarose gel.

4. Single cell clone analysis: Single cells were prepared by digestion of cells with 0.25% trypsin-EDTA. Cells were counted and serially diluted to 2 x 10^4^cells/ml, 5 x 10^2^cells/ml and 5 cells/ml. Next, 200 μl of 5cells/ml was dispensed to each well of 96-well plates using a multichannel pipette. Plates were incubated at 37°C in a 5% CO_2_ incubator.

5. Screening knock-in mutation (N551K and R1398H): Genomic DNA was isolated from single clones. The donor target region was PCR amplified with AmpliTaq Gold 360 master mix (Thermo Fisher) (see PCR primer sequences in S6 Table). PCR amplicons were sequenced using standard Sanger sequencing.

6. Screening KO: Cell lysates were prepared from single cell clones and western blot analysis were used to screen knockout clones.

### Cell culture and NOD2 transfection

LRRK2 WT, LRRK2 DM or LRRK2 KO RAW264.7 cells were maintained in DMEM medium supplemented with 10% fetal bovine serum and 1% streptomycin-penicillin, and incubated in a humidified atmosphere containing 5% CO_2_ at 37°C. The cells were passaged every 3 days. Plasmid pcDNA3.1+/C-(K)DYK-NOD2 encoding Flag tagged human wild-type NOD2 was purchased from GenScript. A plasmid pcDNA3.1+/C-(K)DYK-NOD2 R702W encoding NOD2 R702W variant was generated using a QuickChange XL site-directed mutagenesis kit (Agilent Technologies) according to the manufacturer’s instructions. Primers used for generation of NOD2 R702W mutation were 5’-ggcctggcgccagagcagggcct-3’ and 5’-aggccctgctctggcgccaggcc-3’. The NOD2 R702W mutation was confirmed by Sanger sequencing. Plasmids pcDNA3.1+/C-(K)DYK-NOD2, pcDNA3.1+/C-(K)DYK-NOD2 R702W and pcDNA3.1+/C-(K)DYK empty vector (negative control) were transfected into the three RAW264.7 cells lines using a Neon transfection system (Thermo Fisher Scientific). Briefly, 1 × 10^6^ cells were suspended in 100 μL of buffer R containing 15 μg of plasmid and electroporated at 1,680 V for 20 ms and 1 pulse.

### Preparation of BCG-Russia and *M*. *leprae*

BCG Russia culture was maintained in middlebrook7H9 medium supplemented with 10% ADC, 0.1% Tween 80, and 0.2% glycerol at 37°C on a roller. On the day of infection, appropriate amount of log-phase BCG-Russia were transferred to a 50 ml conical tube and pelleted by centrifugation at 2500 rpm for 6 min. The supernatant was removed, and the cell pellet was washed once with 1x PBS and re-suspended in complete DMEM medium. To break up large aggregates into single cells, the re-suspended BCG was treated in water bath sonication for 20s x 5 times, followed by passing the BCG through a 22 1/2-G needle 8 times. The remaining bacterial clumps were removed by centrifugation for 5min at a centrifugation force of 100g. Bacterial load was determined by plating serial 10-fold dilutions of BCG on Middlebrook 7H10 agar plate (supplemented with 10% OADC) and counting colonies after incubation for at least 3 weeks. Viable *M*. *leprae* was obtained from the National Hansen’s Disease Program, Health Resources and Services Administration, Baton Rouge, LA, USA.

### ROS detection

Cells were seeded in 96-well plates at a concentration of 3×10^4^ cells per well and stimulated with IFN-γ (100ng/ml) for 24 hours and then infected with BCG-Russia or *M*. *leprae* at a MOI of 10:1. At indicated time points following infection, intracellular ROS was detected using ROS-ID total ROS detection kit (Enzo life science) according to the manufacturer’s instruction. For that, cells were carefully washed with 200 μl/well of 1× wash buffer. Following wash buffer removal, 100 μl/well of ROS detection mix (4 μl of 5mM oxidative stress detection reagent /10 ml of 1× wash buffer) was added prior to incubation of plates in a humidified incubator (37°C, 5% CO_2_) for 30 min and reading were acquired at wavelength 488/520nm on a plate reader. The experiment was performed three times, each in triplicate.

### FACS analysis of apoptosis

LRRK2 WT or CRISPR/Cas9-edited RAW264.7 cells (mock transfected, transfected with *NOD2* constructs or empty vector) were separately cultured at a concentration of 6×10^5^/well in a 6-well plate for 16–18 hrs. The cells were then infected with BCG (MOI 10:1) or left uninfected for 24 hours prior to apoptosis detection. The Annexin V staining was done according to the manufacturer’s instruction (Biolegend). Briefly, the cells were first detached from the culture plates and washed twice with 2mL of 1X azide-free and serum/protein-free PBS at RT. The supernatant was discarded. Then, 0.5μl of Zombie Aqua fixable viability dye (ZA-FVD) was added to 100μL of cells in 1X azide-free and serum/protein-free PBS and incubated in dark for 30 minutes at 2°C. After incubation, the cells were washed twice with 1X azide-free PBS+0.2% BSA. The cells were washed once with 1X azide-free PBS+0.2% BSA and then once with 1X Annexin V Binding Buffer (BD Biosciences). The cells were resuspended in 1X Binding Buffer at 2x10^6^ cells/ml. Next, 5μl of Annexin V-APC were added to 100μL of the cell suspension and incubated in dark for 15 minutes at room temperature. After incubation, the cells were washed twice with 2ml of 1X Binding Buffer. The supernatant was discarded. The cells were resuspended in 200μl of 1 X Binding Buffer and immediately collected by flow cytometry with BD FACSCanto II (BD Biosciences). The data was analyzed on FlowJo v10.4.2 (FlowJo, LLC) with viability and Annexin V single stains as FMOs.

### Western Blot analysis

Equivalent amounts of total cellular lysates were separated on 4% to 12% Tris-Glycine gels (Invitrogen) and electrophoretically transferred to polyvinylidene difluoride membranes (Millipore, Bedford, MA). The membranes were blocked with 5% BSA in TBS-T (Tris-buffered saline-0.1% Tween 20) for 1h at room temperature (RT), and then followed by incubation with primary antibodies overnight at 4°C. A rabbit monoclonal antibody against LRRK2 (Abcam) was used at 1:1,000 dilution. A mouse anti-GAPDH monoclonal antibody (Thermo Fisher) was used at a 1:10,000 dilution. A mouse monoclonal antibody against FLAG (Sigma) was used at 1: 2,500. A rabbit anti-phospho-RIP2 (S176) (abm) was used at 1:1,000. After incubation, membranes were washed 5 times for 5min with TBS-T and were further incubated with appropriate secondary antibodies coupled to horseradish peroxidase for 1h RT. Upon extensive washing, the membrane was developed with enhanced chemiluminescence detection reagents (Bio-Rad), followed by imaging using a ChemiDoc Touch imaging system (Bio-Rad).

### Co-immunoprecipitation

For co-immunoprecipitations, 24 hours after electroporation, cells were treated with or without 5μg/ml of N-glycolyl MDP for another 24 hours, and then cells were lysed in Pierce IP lysis/wash buffer supplemented with 1X protease inhibitor mixture (Thermo Fisher scientific). Cell lysates were centrifuged at 13,000xg for 10 min to remove cellular debris. Next, 800μg of the total cell lysates was incubated with 5μg of the rabbit anti-LRRK2 antibody (Abcam) overnight at 4°C with rotation. Then, 500μl of Pierce protein A/G magnetic beads was added and incubated at room temperature for 1h with rotation. The beads were collected with a magnetic stand and washed three times with IP lysis/wash buffer and once with ultrapure water. The proteins bound to the beads were eluted and analyzed by Western blot, which were detected with a mouse anti-LRRK2 (1:1,000; EMD Millipore) or mouse anti-FLAG (1:2,500; Sigma) primary antibody and IP-specific secondary antibody (anti-mouse IgG for IP, Abcam).

### Co-localization with laser confocal microscopy in RAW cells

Transfected or untransfected cells were seeded in 8-well chambers. Twenty-four hours after transfection, cells were treated with or without 5 μg of N-glycolyl MDP 2 for another 24 hours. Cells were fixed with 4% paraformaldehyde, permeabilized with 0.1% Triton X-100. Samples were blocked with blocking buffer [5% BSA, 2.52 mg/ml glycine in PBST (PBS + 0.1% Tween 20)] and incubated with the primary antibodies diluted in blocking buffer–rabbit anti-LRRK2 1:500 and mouse anti-FLAG 1:250 (for NOD2)–overnight at 4°C. Cells were washed with PBS for 3 x 5 minutes and incubated with secondary antibodies prepared in a diluted (1:5) blocking buffer [Alexa Fluor 488 goat anti-mouse IgG (H+L) 1:1000 and Alexa Fluor 594 donkey anti-rabbit IgG (H+L) 1:1000] for 1 hour. Cells were washed with PBS for 3 x 5 minutes and nuclei were stained with DAPI. Images were obtained by confocal microscopy. Colocalization between LRRK2 and NOD2 was measured from 25–30 cells by Zeiss 2012 ZEN confocal software.

### Co-localization analysis in human monocyte-derived macrophages

Primary monocytes were obtained from peripheral blood monocytes from a healthy volunteer and two members of the studied family: the father and one of the twin girls (Fig 1). The blood, once collected, was transferred from the collection tube to a 50ml falcon tube (Corning) and diluted in sterile PBS (1:1) (Thermo Fisher Scientific). Once diluted, 25ml of blood was slowly transferred to another 50ml falcon tube containing 20mL of Ficol Paque (GIBCO) and centrifuged at 800g for 30 min at 25°C without brake. After centrifugation, peripheral blood mononuclear cells (PBMC) were separated in a new 50 ml falcon tube and washed with sterile PBS by centrifugation at 700g for 10 min at 25°C, and again in 20 ml by centrifugation at 500g for 10 min at 25°C. Finally, cells were resuspended in 2ml of RPMI medium supplemented with 2% FBS (GIBCO), 2mM of L-glutamine (L-GLU) (GIBCO) and 100 μg/ml of Penicillin-Streptomycin (PEES) (GIBCO). Cells were counted in a Neubauer chamber and 2x10^6^ PBMC (approximately 2x10^5^ monocytes) were plated per well, in a 24-well plate containing coverslips, in a final volume of 500μl of RPMI medium supplemented by 2% FBS, 2mM of L-GLU and 100 μg/ml of PEES for 2h at 37°C. After 2 hrs, non-adherent cells were gently washed 2 times with warm PBS and then incubated with 1ml of RPMI medium supplemented by 10% FBS, 2mM of L-GLU, 100 μg/mL of PEES and 100ng of GM-CSF (PrepoTech). After 3 days, half of the medium volume was removed and completed with another 500 μl of RPMI medium supplemented by 10% FBS, 2mM of L-GLU, 100μg /ml of PEES and 100ng of GM-CSF. On day 6 the cells were treated with 10ug/ml of N-glycolyl-MDP for another 24 hrs.

After that, coverslips with cells were fixed with 4% paraformaldehyde (Sigma-Aldrich) solution for 24 hrs and then 2% paraformaldehyde solution for another 48h. Then the coverslips were submitted to the immunofluorescence labeling protocol, which consists of a step of 30 minutes in a blocking solution: PBS supplemented by 5% normal goat serum (NGS) (Life technologies ref: PCN5000), 2% BSA (Sigma ref. A9418) and 0.1% triton X-100 (Amersham Biosciences, Ref: 17-115-01). This step was followed by a 1-hr incubation with the primary antibodies against LRRK2 (AB133474) at a dilution of 1:50 and against NOD2 (MA116611) at a dilution of 1:100. After the incubation time with the primary antibodies, the coverslips were washed 3 times in PBS. Then the coverslips were incubated with the secondary antibodies, Alexa 633 Goat anti-Rabbit (ThermoFisher Scientific) (1:500) and Alexa 546 Goat anti-Mouse (ThermoFisher Scientific) (1:400) for 30 minutes. After incubation with the secondary antibodies, coverslips were incubated with DAPI for 2 minutes and then washed 3 times in PBS. Coverslips were mounted on glass slides with ProLong Gold Antifade Mountant (ThermoFisher Scientific) and sealed with Permount Mounting Medium (Fisher Scientific). Images were obtained by confocal microscopy. Colocalization between LRRK2 and NOD2 was measured from 40 cells by LAS X Software (Leica).

### Luciferase reporter assays

Luciferase reporter assays were performed using Dual-Luciferase Reporter Assay System (Promega) according to the manufacturer’s instructions. Briefly, 1×10^6^ LRRK2 WT, DM or KO RAW 264.7 cells were suspended in 100μl of a solution (buffer R) containing 10μg of pcDNA3.1+/C-(K)DYK-NOD2, pcDNA3.1+/C-(K)DYK-NOD2 R702W or empty vector control, 3μg of *NF-κB firefly-*Luc plasmid, and 1μg of Renilla-Luc plasmid pRL-TK. Cells were transfected by electroporation using the Neon Transfection System (Thermo Fisher Scientific) at 1,680V for 20 ms and 1 pulse. Cells from each transfection reaction were plated in a 24-well plate with 1×10^5^ cells per well. Twenty-four hours after electroporation, cells were treated with 10μg/ml of N-glycolyl MDP for another 24 hours or left untreated; following this, cell lysates were prepared by using Passive Lysis Buffer (Promega). Luciferase activity was determined from a 20-μL cell lysates and measured on the microplate reader. Firefly luciferase activity was normalized to *Renilla* luciferase activity.

### Cytokine and chemokine measurements

For cytokine and chemokine measurements, twenty-four hours after electroporation cells were incubated with or without BCG-Russia (MOI 10:1) for another 24 hours. Cell culture supernatants were collected and centrifuged to remove debris. Cytokines and Chemokines were detected using Milliplex Map (EMD Millipore, St. Charles, MO, USA) multiplex magnetic bead-based antibody detection kits according to the manufacturer’s instructions. A 6-plex premixed kit was used, which included analytes IL-1β, IL-6, IL-10, IL-12 (p40), MCP-1 and TNF. Assay was read using xPONENT 3.1 acquisition software and MAGPIX instrument (Luminex Corporation, Toronto, ON, Canada). Data was analyzed using Mulliplex Analyst software v4.2 (EMD Millipore) and presented as picogram of cytokine per milliliter of supernatants (pg/ml).

### Statistical analysis

Statistical analysis was conducted using GraphPad Prism 5 (GraphPad Software, California USA, www.graphpad.com). Kinetics of ROS production were analyzed by comparing the mutant cells with the wild-type cells at each timepoint using two-way ANOVA with Bonferroni correction. In apoptosis, NF-κB activation and cytokine secretion assays, one-way ANOVA and *post hoc* t test with Bonferroni correction was used to compare means between two groups. Co-localization analysis was done using one-way ANOVA, comparing each condition with the control using Kruskal-Wallis test. Adjusted *P* < 0.05 was used as significance threshold, which is represented by asterisks in the figures. Data visualization for bar plots and box plots was done using ggplot2 package in R version 3.6.3 (https://www.R-project.org/" https://www.R-project.org/).

## Supporting information

S1 Fig Custom filtering approaches for candidate variants identification from WGS data in the studied family. First, variants were selected based on their location and impact in protein-coding genes (shown on top). Then, seven different filtering approaches were applied (approaches #1 to #7). These filtering steps were based i) on the variant frequencies in public databases, ii) on the model of inheritance and iii) on the age-at-diagnosis of the leprosy-affected family members. Specifically, recessive (#1 to #4) and dominant (#5 to #7) models were tested based on the presence of the variant in all affected family members (#1 and #5), only in the cases younger than 30 years (#2 and #6) and only in the early-onset twins with less than 2 years (#3, #4 and #7). In the pedigree, men and women are represented by boxes and circles, respectively. Leprosy patients, regardless of the subtype, are indicated by filled symbols. Monozygocity is represented by a triangle. The number zero in blue represents the reference allele and the number one in red corresponds to the variant. The sample ID is the same as Fig 1. The lists of candidate variants detected using these approaches are presented in S2 and S3 Tables. 1000G: The 1000 genome consortium database; DSV: deletion structural variant; ExAC: The Exome Aggregation consortium database; Indel: insertion/deletion; SNV: single nucleotide variant.
(TIF)

S2 Fig All missense variants in *LRRK2* and *NOD2* genes detected by WGS in the studied family. The *LRRK2* missense variants found in the family were rs2256408 (R50H), rs7308720 (N551K), rs78365431 (Q1111H), rs7133914 (R1398H), rs11564148 (S1647T) and rs3761863 (M2397T). Four missense variants were detected in *NOD2*, which were rs2066842 (P268S), rs104895438 (A612T), rs2066844 (R702W) and rs5743278 (A725G). Among these variants, *LRKK2* N551K and R1398H passed filtering approaches #2 and #3, respectively; while *NOD2* R702W passed filtering approach #6 (see filtering approaches in S1 Fig). The alternative allele from the three candidate variants that passed filtering are shown in bold. No coding indels were detected in *LRRK2* and *NOD2* genes in the WGS data from the studied family. Men and women are represented by boxes and circles, respectively. Leprosy patients, regardless of the subtype, are indicated by filled symbols, while unknown phenotype is indicated by symbol with diagonal stripes. Monozygosity is represented by a horizontal line linking siblings. The sample ID is the same as Fig 1.
(TIF)

S3 Fig Population structure of the studied family using principal component analysis based on 237,150 variants from the WGS data. Each dot represents an individual, including the six family members from the present study and 2,504 unrelated individuals from the 1000 Genomes Consortium representing the five super populations: African/African American (AFR), Admixed American/Latin (AMR), East Asian (EAS), European (EUR) and South Asian (SAS). **(A)** First and second components are plotted on the *x* and *y* axis, respectively. **(B)** First and third components are plotted on the *x* and *y* axis, respectively.
(TIF)

S4 Fig Effect of LRRK2 R1398H genotype on respiratory burst and apoptosis in response to BCG infection. **(A)** Kinetics of reactive oxygen species (ROS) production upon BCG challenge in RAW cells expressing wild-type (WT), 1398R/H heterozygous (HET) or 1398H/H homozygous (HOM) LRRK2 proteins. Box plots presents the results at each time point showing the ROS measurement on the *y* axis and the genotype groups on the *x* axis. **(B)** Effect of LRRK2 WT, R1398H HET and HOM on apoptosis in response to BCG. Percentage of total apoptotic cells, including cells with early and late apoptosis, was derived for non-infected (left box plot) and BCG-infected cells (right box plot). **(A-B)** LRRK2 R1398H HET and HOM cells were compared to WT using (**A**) two-way ANOVA (ROS, *P* = 0.0004) and (**B**) one-way ANOVA (Apoptosis, *P* < 0.001), followed by post-hoc t test with Bonferroni correction. Pairwise comparisons between WT and HET or HOM are represented by the blue and red lines on top of the box plots, respectively. A linear regression model was used to analyze the dose-dependent effect of LRRK2 R1398H minor allele on: (**A**) ROS production by time point and (**B**) apoptosis by infection status (WT→HET→HOM). Results from the trend tests are shown in black with the regression lines presented as dotted lines and the *P*-values shown below the box plots. **** *P* < 0.0001; ** 0.001 ≤ *P* < 0.01; ns: non-significant. BCG: Bacillus Calmette–Guérin.
(TIF)

S5 Fig Flow cytometry of the BCG-induced apoptosis analysis of NOD2-transfected cells with different LRRK2 genotypes. LRRK2 Wild-type (WT), CRISPR/Cas-edited LRRK2 double-mutant (DM, N551K+R1398H) and LRRK2 knock-out (KO) RAW264.7 cell lines were transfected with plasmids expressing NOD2 WT, NOD2 mutant (R702W) or an empty vector [pcDNA3.1+/C-(K)DYK] as a control. Twenty-four hours post-transfection, cells were left uninfected or infected with live bacillus Calmette–Guérin (BCG)-Russia (MOI 10:1) for another 24 hours. Cells were then harvested, stained with Annexin V/ZA-FVD, and analyzed by flow cytometry for apoptosis. The illustrated result is a representative of two independent experiments with similar results (done in triplicates).
(TIF)

S6 Fig Representative confocal image of colocalization of ectopically expressed NOD2 with LRRK2. Colocalization of LRRK2 wild-type (WT) and CRISPR/Cas-edited LRRK2 double-mutant (DM, N551K+R1398H) in cells transfected with plasmids expressing **(A)** NOD2 WT and **(B)** NOD2 R702W. **(C)** LRRK2 KO cells (top panel) and untransfected LRRK2 WT cells (bottom panel) were used as a negative control for antibody specificity. **(A-D)** RAW264.7 cell lines with the three LRRK2 genotypes were transfected with NOD2 plasmid and a LRRK2 WT cell line was kept untransfected. Twenty-four hours after electroporation, the transfected and untransfected cells were treated with or without 10 μg/mL of N-glycolyl MDP for another 24 hours. Cells were fixed with 4% paraformaldehyde, permeabilized and double stained for LRRK2 and NOD2 with rabbit anti-LRRK2 (1:500) and mouse anti-FLAG (1:250) antibodies. Nuclei were stained with DAPI. Images were obtained by confocal microscopy. **(D)** Colocalization between LRRK2 and NOD2 was measured from 25–30 cells by Zeiss 2012 ZEN confocal software.
(TIF)

S7 Fig Effects of LRRK2 variants on MDP-induced phosphorylation of RIP2 at Ser 176. LRRK2 Wild-type (WT), CRISPR/Cas-edited LRRK2 double-mutant (DM, N551K+R1398H) and LRRK2 knock-out (KO) RAW264.7 cell lines were transfected with plasmids expressing NOD2 WT, NOD2 mutant (R702W) or an empty vector [pcDNA3.1+/C-(K)DYK] as a control. Twenty-four hours post-transfection, cells were left untreated or treated with different concentrations of N-glycolyl MDP as indicated, for another 24 hours. Cell lysates were prepared and the phosphorylation of RIP2 (p-RIP2) in the transfected cell lines was analyzed by immunoblotting with a specific antibody against RIP2 when phosphorylated at Ser 176. GAPDH was used as a loading control.
(TIF)

S1 Table Summary of whole genome sequencing data and mapping quality control of six samples from the studied family. (DOCX)

S2 Table Candidate single nucleotide variants (SNVs) and short Indels identified in the studied family by applying the custom filtering approaches shown in S1 Fig. (DOCX)

S3 Table Candidate deletion structural variants (DSVs) identified in the studied family by applying the custom filtering approaches shown in S1 Fig. (DOCX)

S4 Table Minor allele frequency (MAF) in GnomAD super-populations and estimated genotype frequencies of the LRRK2 and NOD2 variants found in the twins. (DOCX)

S5 Table Genes previously associated with leprosy in GWAS or target association studies up to January 2023 that were included in the leprosy gene analysis from the WGS data (S1 Fig). (DOCX)

S6 Table Oligonucleotides used in the CRISPR/Cas construct of Lrrk2 variants. (DOCX)

S1 Data Excel file containing, in separate sheets, the underlying numerical data for Figs 2B–2E, 3B, 3C, 4C, 4F, 5A, 5B, S4A, S4B and S6. (XLSX)

S1 Method Whole exome sequencing for variant validation. (DOCX)
