# Supplementary figures and images for: Allele-dependent interaction of LRRK2 and NOD2 in leprosy
Source: PLoS Pathog. 2023 Mar 27;19(3):e1011260. doi: 10.1371/journal.ppat.1011260 (PMC10079233; doi:10.1371/journal.ppat.1011260)

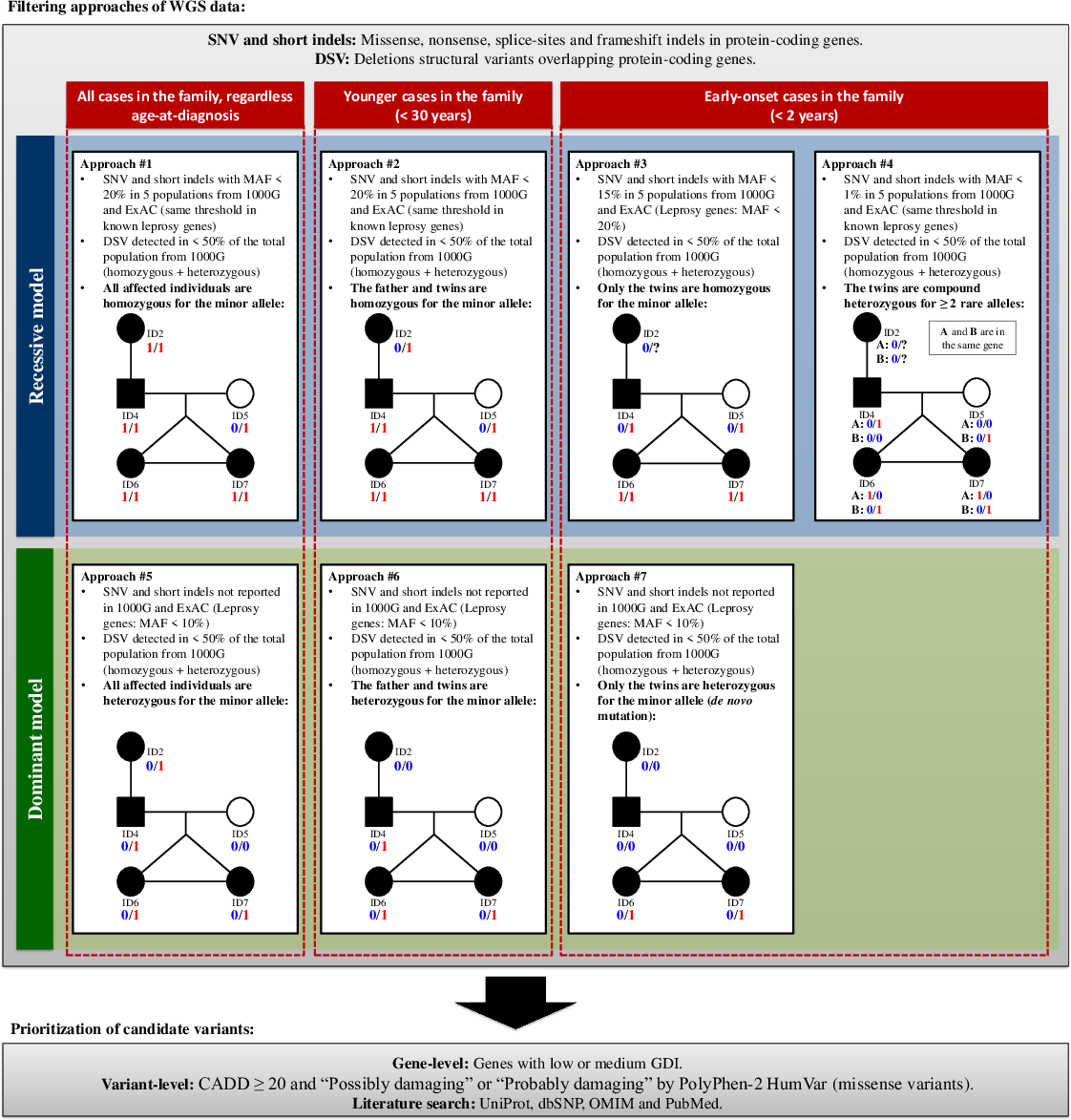

Supplement: S1 Fig — First, variants were selected based on their location and impact in protein-coding genes (shown on top). Then, seven different filtering approaches were applied (approaches #1 to #7). These filtering steps were based i) on the variant frequencies in public databases, ii) on the model of inheritance and iii) on the age-at-diagnosis of the leprosy-affected family members. Specifically, recessive (#1 to #4) and dominant (#5 to #7) models were tested based on the presence of the variant in all affected family members (#1 and #5), only in the cases younger than 30 years (#2 and #6) and only in the early-onset twins with less than 2 years (#3, #4 and #7). In the pedigree, men and women are represented by boxes and circles, respectively. Leprosy patients, regardless of the subtype, are indicated by filled symbols. Monozygocity is represented by a triangle. The number zero in blue represents the reference allele and the number one in red corresponds to the variant. The sample ID is the same as Fig 1. The lists of candidate variants detected using these approaches are presented in S2 and S3 Tables. 1000G: The 1000 genome consortium database; DSV: deletion structural variant; ExAC: The Exome Aggregation consortium database; Indel: insertion/deletion; SNV: single nucleotide variant. (TIF) [file ppat.1011260.s001.tif]

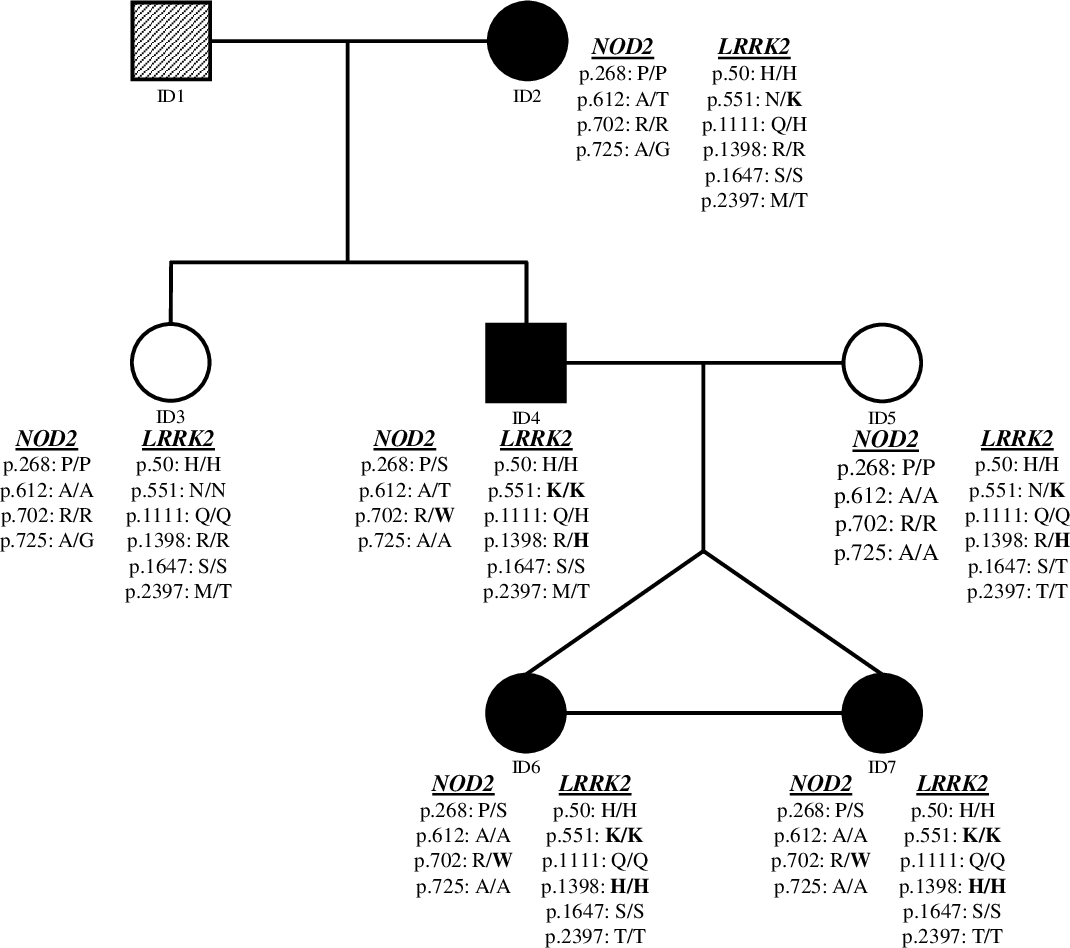

Supplement: S2 Fig — The LRRK2 missense variants found in the family were rs2256408 (R50H), rs7308720 (N551K), rs78365431 (Q1111H), rs7133914 (R1398H), rs11564148 (S1647T) and rs3761863 (M2397T). Four missense variants were detected in NOD2, which were rs2066842 (P268S), rs104895438 (A612T), rs2066844 (R702W) and rs5743278 (A725G). Among these variants, LRKK2 N551K and R1398H passed filtering approaches #2 and #3, respectively; while NOD2 R702W passed filtering approach #6 (see filtering approaches in S1 Fig). The alternative allele from the three candidate variants that passed filtering are shown in bold. No coding indels were detected in LRRK2 and NOD2 genes in the WGS data from the studied family. Men and women are represented by boxes and circles, respectively. Leprosy patients, regardless of the subtype, are indicated by filled symbols, while unknown phenotype is indicated by symbol with diagonal stripes. Monozygosity is represented by a horizontal line linking siblings. The sample ID is the same as Fig 1. (TIF) [file ppat.1011260.s002.tif]

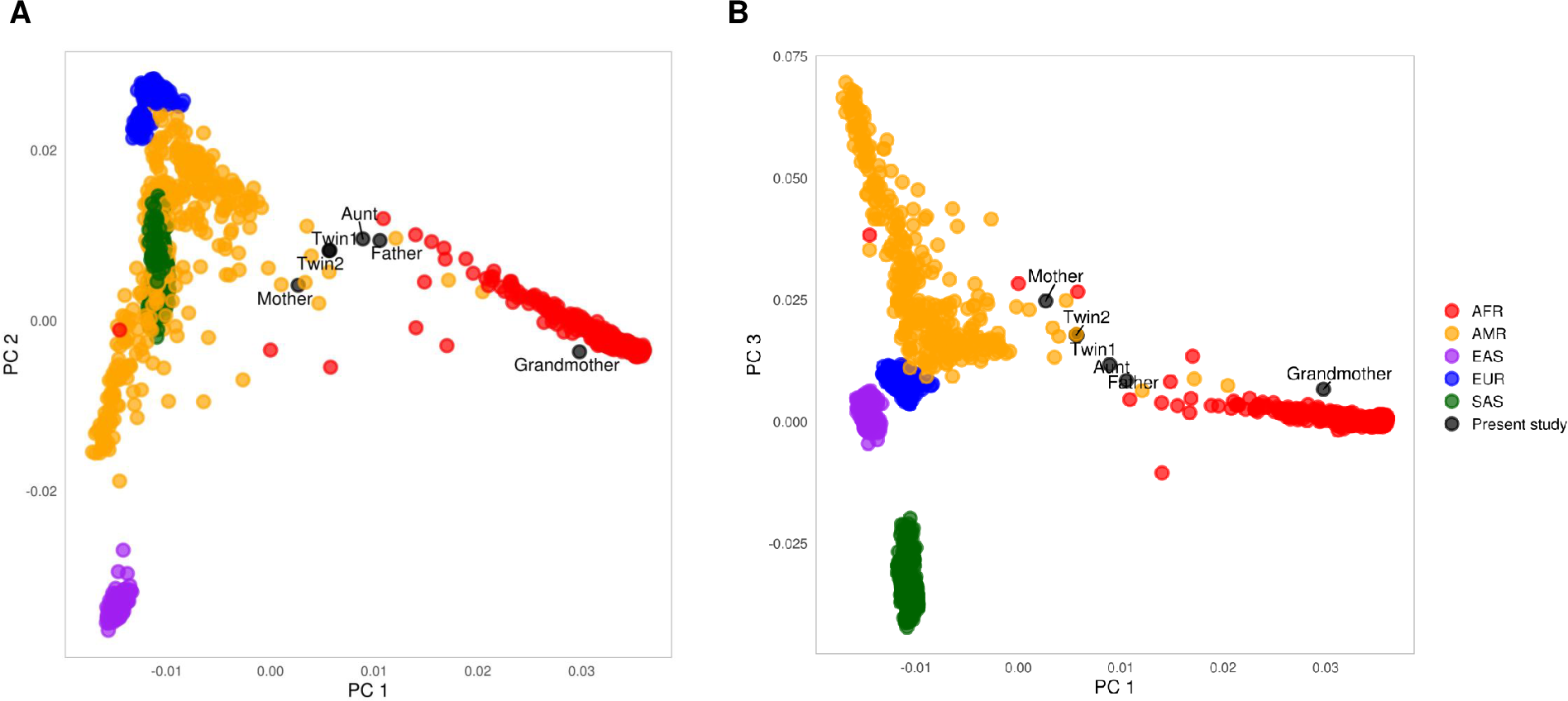

Supplement: S3 Fig — Each dot represents an individual, including the six family members from the present study and 2,504 unrelated individuals from the 1000 Genomes Consortium representing the five super populations: African/African American (AFR), Admixed American/Latin (AMR), East Asian (EAS), European (EUR) and South Asian (SAS). (A) First and second components are plotted on the x and y axis, respectively. (B) First and third components are plotted on the x and y axis, respectively. (TIF) [file ppat.1011260.s003.tif]

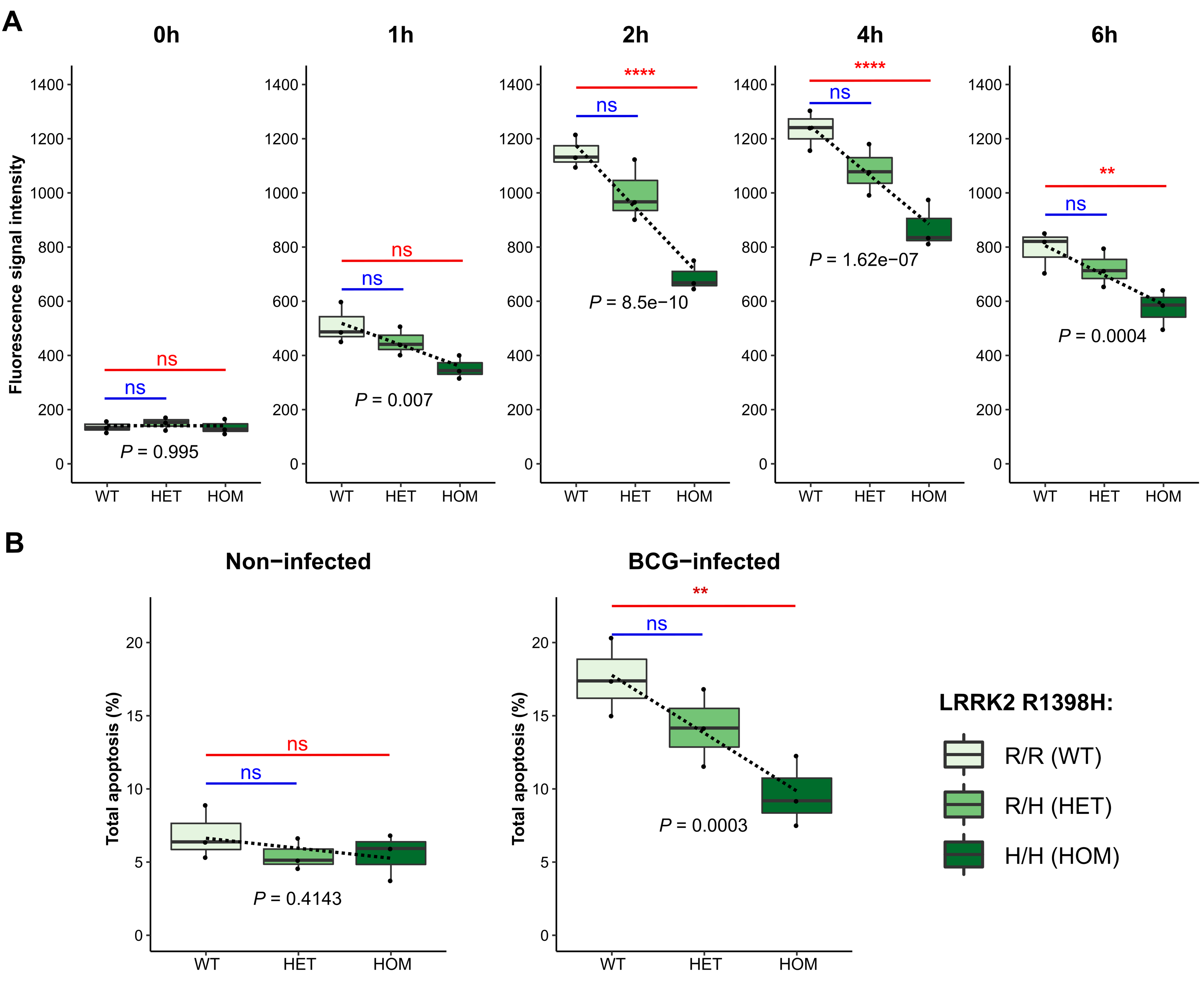

Supplement: S4 Fig — (A) Kinetics of reactive oxygen species (ROS) production upon BCG challenge in RAW cells expressing wild-type (WT), 1398R/H heterozygous (HET) or 1398H/H homozygous (HOM) LRRK2 proteins. Box plots presents the results at each time point showing the ROS measurement on the y axis and the genotype groups on the x axis. (B) Effect of LRRK2 WT, R1398H HET and HOM on apoptosis in response to BCG. Percentage of total apoptotic cells, including cells with early and late apoptosis, was derived for non-infected (left box plot) and BCG-infected cells (right box plot). (A-B) LRRK2 R1398H HET and HOM cells were compared to WT using (A) two-way ANOVA (ROS, P = 0.0004) and (B) one-way ANOVA (Apoptosis, P < 0.001), followed by post-hoc t test with Bonferroni correction. Pairwise comparisons between WT and HET or HOM are represented by the blue and red lines on top of the box plots, respectively. A linear regression model was used to analyze the dose-dependent effect of LRRK2 R1398H minor allele on: (A) ROS production by time point and (B) apoptosis by infection status (WT→HET→HOM). Results from the trend tests are shown in black with the regression lines presented as dotted lines and the P-values shown below the box plots. **** P < 0.0001; ** 0.001 ≤ P < 0.01; ns: non-significant. BCG: Bacillus Calmette–Guérin. (TIF) [file ppat.1011260.s004.tif]

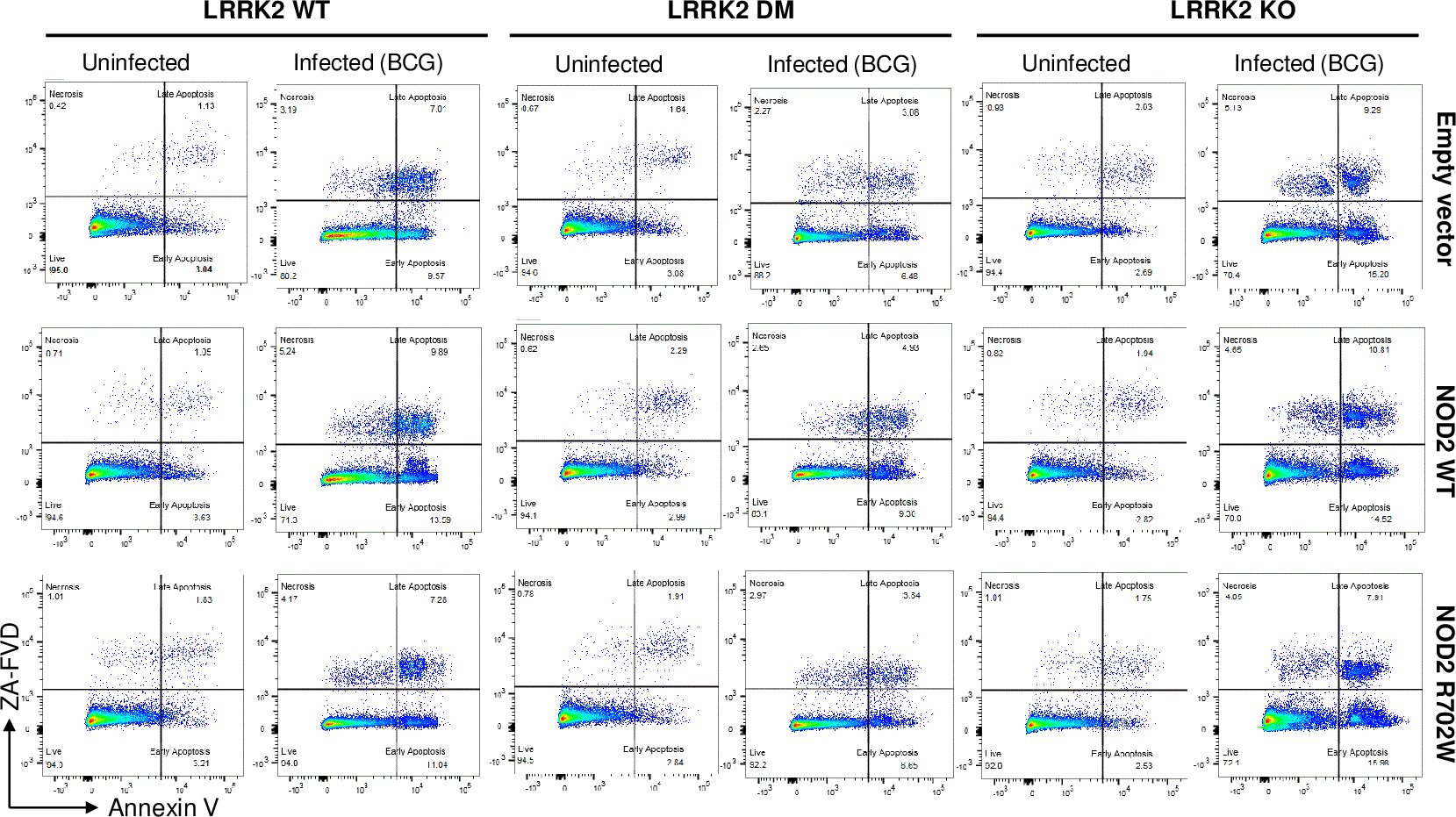

Supplement: S5 Fig — LRRK2 Wild-type (WT), CRISPR/Cas-edited LRRK2 double-mutant (DM, N551K+R1398H) and LRRK2 knock-out (KO) RAW264.7 cell lines were transfected with plasmids expressing NOD2 WT, NOD2 mutant (R702W) or an empty vector [pcDNA3.1+/C-(K)DYK] as a control. Twenty-four hours post-transfection, cells were left uninfected or infected with live bacillus Calmette–Guérin (BCG)-Russia (MOI 10:1) for another 24 hours. Cells were then harvested, stained with Annexin V/ZA-FVD, and analyzed by flow cytometry for apoptosis. The illustrated result is a representative of two independent experiments with similar results (done in triplicates). (TIF) [file ppat.1011260.s005.tif]

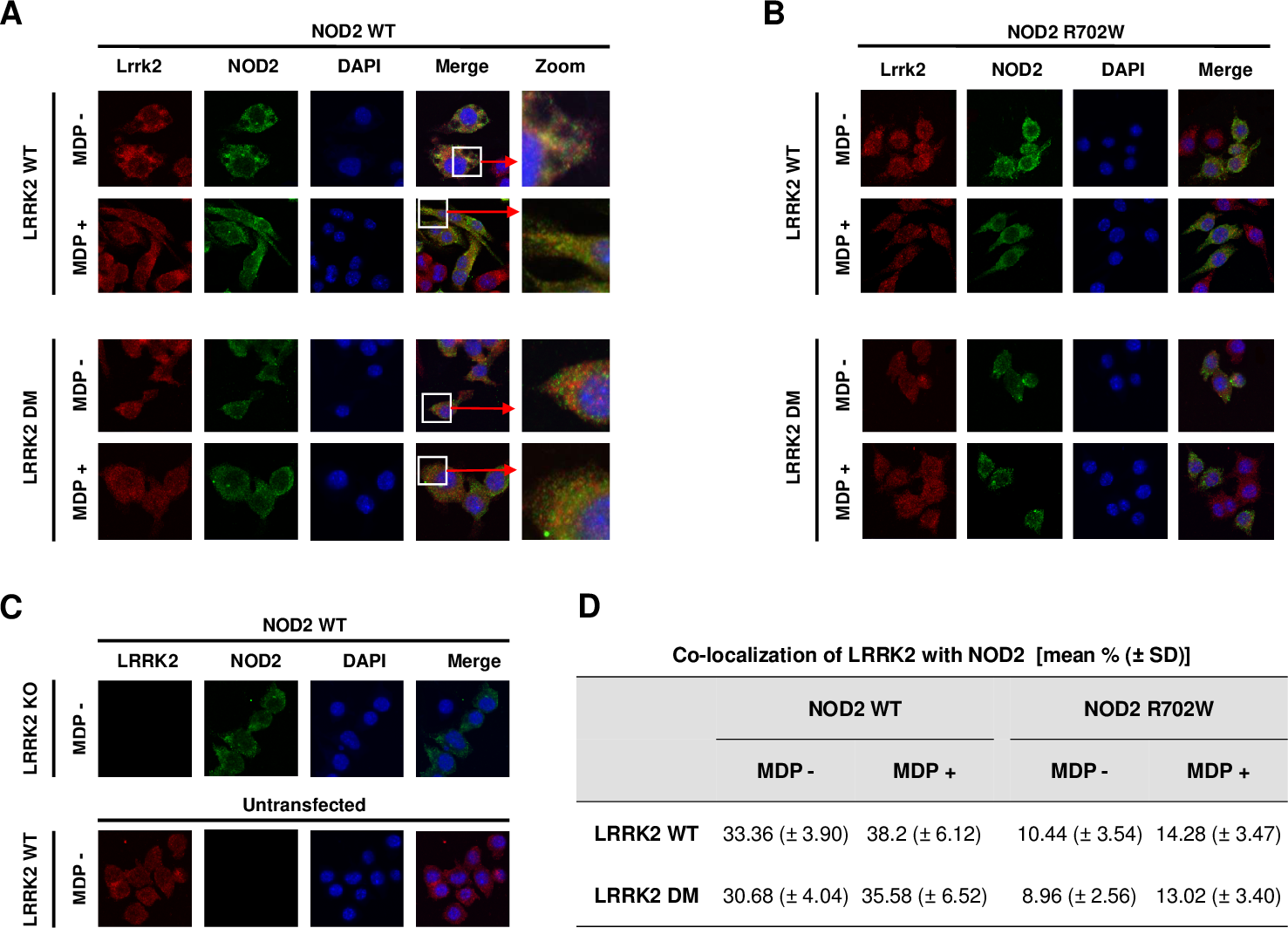

Supplement: S6 Fig — Colocalization of LRRK2 wild-type (WT) and CRISPR/Cas-edited LRRK2 double-mutant (DM, N551K+R1398H) in cells transfected with plasmids expressing (A) NOD2 WT and (B) NOD2 R702W. (C) LRRK2 KO cells (top panel) and untransfected LRRK2 WT cells (bottom panel) were used as a negative control for antibody specificity. (A-D) RAW264.7 cell lines with the three LRRK2 genotypes were transfected with NOD2 plasmid and a LRRK2 WT cell line was kept untransfected. Twenty-four hours after electroporation, the transfected and untransfected cells were treated with or without 10 μg/mL of N-glycolyl MDP for another 24 hours. Cells were fixed with 4% paraformaldehyde, permeabilized and double stained for LRRK2 and NOD2 with rabbit anti-LRRK2 (1:500) and mouse anti-FLAG (1:250) antibodies. Nuclei were stained with DAPI. Images were obtained by confocal microscopy. (D) Colocalization between LRRK2 and NOD2 was measured from 25–30 cells by Zeiss 2012 ZEN confocal software. (TIF) [file ppat.1011260.s006.tif]

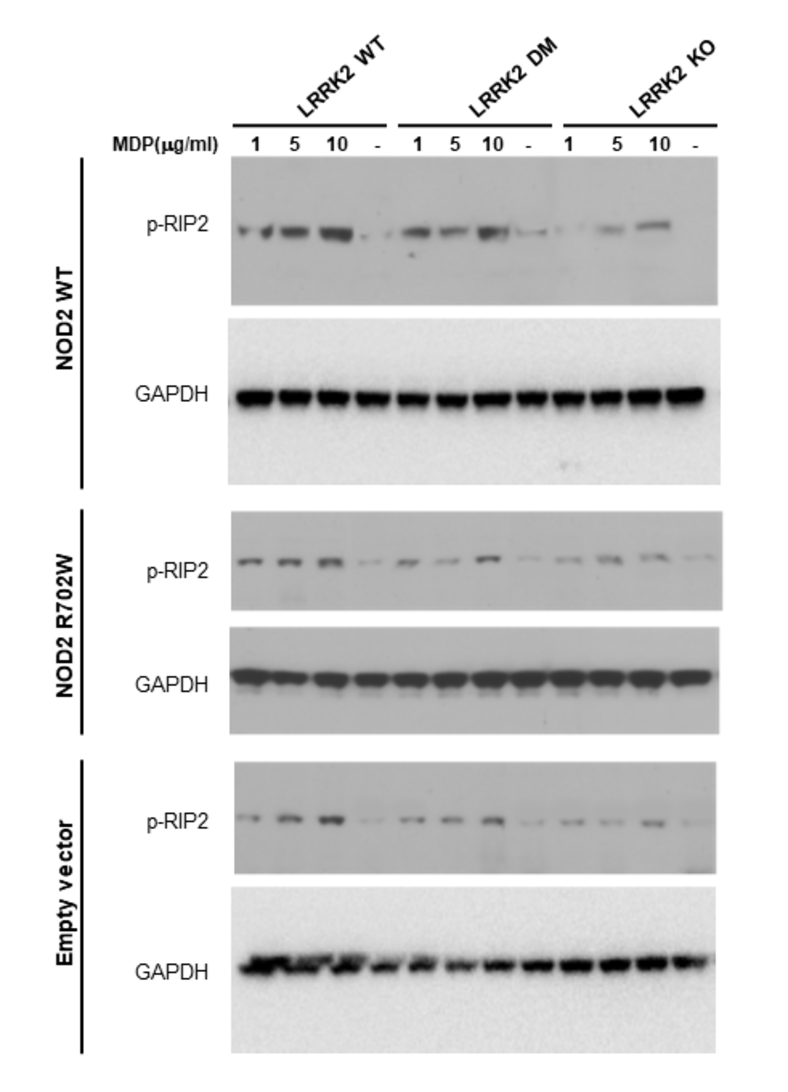

Supplement: S7 Fig — LRRK2 Wild-type (WT), CRISPR/Cas-edited LRRK2 double-mutant (DM, N551K+R1398H) and LRRK2 knock-out (KO) RAW264.7 cell lines were transfected with plasmids expressing NOD2 WT, NOD2 mutant (R702W) or an empty vector [pcDNA3.1+/C-(K)DYK] as a control. Twenty-four hours post-transfection, cells were left untreated or treated with different concentrations of N-glycolyl MDP as indicated, for another 24 hours. Cell lysates were prepared and the phosphorylation of RIP2 (p-RIP2) in the transfected cell lines was analyzed by immunoblotting with a specific antibody against RIP2 when phosphorylated at Ser 176. GAPDH was used as a loading control. (TIF) [file ppat.1011260.s007.tif]
