# Supplementary material for: Allele-dependent interaction of LRRK2 and NOD2 in leprosy
Source: PLoS Pathog. 2023 Mar 27;19(3):e1011260. doi: 10.1371/journal.ppat.1011260 (PMC10079233; doi:10.1371/journal.ppat.1011260)
Supplement: S1 Table — (DOCX) [file ppat.1011260.s008.docx]

**S1 Table.** Summary of whole genome sequencing data and mapping quality control of six samples from the studied family.

| **Parameters** | **ID2** | **ID3** | **ID4** | **ID5** | **ID6** | **ID7** | **Average** |  |
| --- | --- | --- | --- | --- | --- | --- | --- | --- |
| No. paired-end reads (millions) | 471 | 416 | 350 | 520 | 437 | 526 | **453** |  |
| Mapped reads | 99.1% | 98.9% | 99.2% | 99.4% | 99.1% | 99.4% | **99.2%** |  |
| Mean Mapping Quality | 51.0 | 51.0 | 51.2 | 51.1 | 51.0 | 51.0 | **51.0** |  |
| Overlapping read pairs | 4.06% | 5.84% | 5.08% | 3.78% | 4.05% | 3.80% | **4.4%** |  |
| Duplicated reads | 12.2% | 12.2% | 11.1% | 12.1% | 10.8% | 11.3% | **11.6%** |  |
| Coverage (mean X ± SD)* | 32 ± 12 | 28 ± 11 | 25 ± 10 | 35 ± 13 | 30 ± 12 | 36 ± 13 | **31 ± 12** |  |
| Genome fraction with ≥ 10X* | 91.4% | 91.3% | 91.5% | 91.4% | 91.3% | 91.4% | **91.4%** |  |
| Genome fraction with ≥ 20X* | 90.3% | 89.3% | 82.0% | 90.7% | 90.0% | 90.8% | **88.8%** |  |
| Genome fraction with ≥ 30X* | 72.0% | 56.7% | 32.1% | 83.5% | 68.1% | 85.3% | **66.3%** |  |
| * Overlapping read pairs and duplicated reads were ignored. | | | | | | | | |
| No.: number; SD: standard deviation; X: times/folds. | | | | | | | | |
