# Supplementary material for: Allele-dependent interaction of LRRK2 and NOD2 in leprosy
Source: PLoS Pathog. 2023 Mar 27;19(3):e1011260. doi: 10.1371/journal.ppat.1011260 (PMC10079233; doi:10.1371/journal.ppat.1011260)
Supplement: S2 Table — (DOCX) [file ppat.1011260.s009.docx]

**S2 Table**. Candidate single nucleotide variants (SNVs) and short Indels identified in the studied family by applying the custom filtering approaches shown in S1 Fig.

| **Gene** | **GDI ^a^** | **Chr** | **Position ^b^** | **Ref** | **Alt** | **Type** | **rsID ^a^** | **MAF**  **(ExAC ALL)** | **AA Change** | **Scaled CADD ^a^** | **PolyPhen-2 HumVar ^a^** | **Prioritized ^c^** |
| --- | --- | --- | --- | --- | --- | --- | --- | --- | --- | --- | --- | --- |
| **Approach #1: Recessive model - All cases in the family, regardless of age-at-diagnosis**  **(Grandmother, father and twins are homozygous for the variant).** | | | | | | | | | | | | |
| None |  |  |  |  |  |  |  |  |  |  |  |  |
| **Approach #2: Recessive model - Younger cases in the family**  **(Father and twins are homozygous for the variant).** | | | | | | | | | | | | |
| *RNH1* | Medium | 11 | 499120 ^d^ | G | A | Missense | rs17585 | 11.39% | P170L | 16.60 | Benign | No |
| *LRRK2* | Medium | 12 | 40657700 ^d^ | C | G | Missense | rs7308720 | 8.61% | N551K | 24.1 | Probably damaging | Yes |
| *CD68* | Medium | 17 | 7484101 ^d^ | C | A | Missense | rs9901673 | 15.45% | Q254K | 12.1 | Benign | No |
| *MPDU1* | Medium | 17 | 7490810 ^d^ | G | A | Missense | rs10852891 | 15.40% | A229T | 20.6 | Benign | No |
| *SALL4* | Medium | 20 | 50406630 ^d^ | T | G | Missense | rs6091375 | 5.10% | I798L | 7.489 | Benign | No |
| **Approach #3: Recessive model - Early-onset cases in the family**  **(Only the twins are homozygous for the variant).** | | | | | | | | | | | | |
| *ZNF678* | Medium | 1 | 227843003 | G | A | Missense | rs61740826 | 2.09% | C406Y | 22.4 | Probably damaging | Yes |
| *LRP1B* | Medium | 2 | 141242918 ^d^ | T | C | Missense | rs34488772 | 4.78% | Q3140R | 0.121 | Benign | No |
| *PRIMPOL* | Medium | 4 | 185580557 ^d^ | A | G | Missense | rs74696256 | 0.90% | T82A | 22.5 | Benign | No |
| *ROS1* | High | 6 | 117710661 ^d^ | T | C | Missense | rs28639589 | 4.59% | I537M | 17.8 | Benign | No |
| *LRRK2* | Medium | 12 | 40702911 ^d^ | G | A | Missense | rs7133914 | 8.41% | R1398H | 23.2 | Possibly damaging | Yes |
| *STAB2* | Medium | 12 | 103988285 ^d^ | A | G | Missense | rs17034186 | 1.16% | I110V | 17.34 | Benign | No |
| *SOS2* | Medium | 14 | 50655307 ^d^ | C | T | Missense | rs61755579 | 1.85% | A208T | 23 | Benign | No |
| *SMPD3* | Medium | 16 | 68395522 ^d^ | C | T | Missense | rs71395853 | 3.87% | C617Y | 18.13 | Benign | No |
| *DHX33* | Medium | 17 | 5354204 ^d^ | G | C | Missense | rs11653658 | 5.59% | H483D | 17.74 | Benign | No |
| *HRH4* | Medium | 18 | 22057204 ^d^ | C | G | Missense | rs58154316 | 1.08% | S284C | 16 | Probably damaging | No |
| *ACP5* | Medium | 19 | 11687195 | C | T | Missense | rs2229531 | 9.66% | V200M | 22.0 | Possibly damaging | Yes |
| *ACP5* | Medium | 19 | 11687351 ^d^ | C | T | Missense | rs2305799 | 10.34% | V148M | 15.69 | Benign | No |
| *LYL1* | Medium | 19 | 13211843 | C | T | Missense | rs117072928 | 8.93% | R48Q | 1.86 | Benign | No |
| *KLK8* | Medium | 19 | 51503285 ^d^ | C | T | Missense | rs16988799 | 4.64% | V154I | 13.99 | Benign | No |
| *DIDO1* | Medium | 20 | 61512185 | G | C | Missense | rs41282984 | 6.40% | S1708C | 10.96 | Benign | No |
| *SLC17A9* | Medium | 20 | 61598731 | C | T | Missense | rs7271712 | 2.65% | T397M | 24.2 | Possibly damaging | Yes |
| *PRDM15* | Medium | 21 | 43221797 ^d^ | G | C | Missense | rs2236695 | 3.83% | T1376S | 16.96 | Benign | No |
| *TCF20* | Medium | 22 | 42607817 ^d^ | C | T | Missense | rs17002890 | 0.99% | M1165I | 22.8 | Benign | No |
| **Approach #4: Recessive model - Early-onset cases in the family**  **(The twins are compound heterozygous).** | | | | | | | | | | | | |
| *FAT1* | High | 4 | 187524714 ^d^ | C | T | Missense | rs192691397 | 0.05% | V3656I | 16.21 | Benign | No |
|  |  |  | 187530423 ^d^ | T | C | Missense | rs138364727 | 0.007% | I3374V | 23.9 | Probably damaging |  |
|  |  |  | 187540374 ^d^ | C | T | Missense | rs370340394 | 0.001% | A2456T | 6.944 | Benign |  |
| *TNXB* | High | 6 | 32049373 | C | T | Missense | - | 0.005% | V1272M | 14.38 | Benign | No |
|  |  |  | 32063558 | C | T | Missense | rs201146825 | 0.007% | G691D | 23.7 | Probably damaging |  |
| *EYS* | High | 6 | 65300527 | C | T | Missense | rs145274061 | 0.02% | D1745N | 8.095 | Benign | No |
|  |  |  | 66005791 | C | T | Missense | - | - | G663E | 14.67 | Benign |  |
| **Approach #5: Dominant model - All cases in the family, regardless of age-at-diagnosis**  **(Grandmother, father and twins are heterozygous for the variant).** | | | | | | | | | | | | |
| *PPP4R2* | Medium | 3 | 73114043 ^d^ | C | T | Missense | - | - | P227S | 17.9 | Benign | No |
| *CP** | Medium | 3 | 148905977 ^d^ | T | C | Missense | - | - | K576E | 26.6 | Possibly damaging | Yes |
| *SOWAHB* | Medium | 4 | 77816977 ^d^ | G | C | Missense | - | - | L676V | 24.6 | Possibly damaging | Yes |
| *SYNPO2** | High | 4 | 119947864 ^d^ | G | A | Missense | - | - | E114K | 22.1 | Benign | No |
| *UGT3A1* | Medium | 5 | 35957503 ^d^ | C | T | Missense | - | - | A288T | 8.7 | Benign | No |
| *HLA-DRB1* | High | 6 | 32552091 ^d^ | G | C | Missense | rs569286159 | 2.67% | F55L | 1.916 | Benign | No |
| *MCM9** | Medium | 6 | 119147418 ^d^ | G | A | Missense | - | - | T618I | 25.6 | Possibly damaging | Yes |
| *CCDC34* | Medium | 11 | 27384674 | C | A | Missense | - | - | R23I | 18.45 | Benign | No |
| *LEMD3* | Medium | 12 | 65563747 | G | A | Missense | - | - | G124D | 16.9 | Benign | No |
| *ERN1* | Medium | 17 | 62130282 ^d^ | T | C | Missense | - | - | K704R | 12.88 | Benign | No |
| *CDH20** | Medium | 18 | 59217360 ^d^ | G | A | Missense | - | - | D600N | 8.2 | Benign | No |
| *SUV39H1* | Medium | X | 48564979 | C | T | Missense | - | - | R356W | 17.12 | Benign | No |
| *ZNF185* | Medium | X | 152113882 ^d^ | C | G | Missense | - | - | A459G | 10.8 | Benign | No |
| **Approach #6: Dominant model - Younger cases in the family**  **(Father and twins are heterozygous for the variant).** | | | | | | | | | | | | |
| *PRKACB* | Medium | 1 | 84650810 ^d^ | G | T | Stop-gain | - | - | E122X | 37 | - | Yes |
| *IQGAP3* | High | 1 | 156521798 ^d^ | T | A | Missense | - | - | Q513L | 17.8 | Benign | No |
| *ASTN1* | Medium | 1 | 176833514 ^d^ | T | G | Missense | - | - | D1264A | 20.1 | Benign | No |
| *CR1* | Medium | 1 | 207785097 ^d^ | A | G | Missense | - | - | E1674G | 21.8 | Possibly damaging | Yes |
| *FSIP2* | High | 2 | 186666910 ^d^ | C | G | Missense | - | - | H4382D | 15 | - | No |
| *NBEAL2* | High | 3 | 47037443 ^d^ | G | A | Missense | - | - | V685M | 22.0 | Possibly damaging | No |
| *OTOP1* | Medium | 4 | 4199651 ^d^ | T | A | Missense | - | - | M304L | 0.174 | Benign | No |
| *SLC25A25* | Medium | 9 | 130830773 | G | A | Missense | - | - | V59I | 21.1 | Benign | No |
| *PDCD11* | High | 10 | 105201712 ^d^ | G | A | Missense | - | - | E1563K | 23 | Benign | No |
| *ADAL* | Medium | 15 | 43627339 ^d^ | A | G | Missense | - | - | T12A | 16.0 | Benign | No |
| *CCPG1* | Medium | 15 | 55670559 ^d^ | C | A | Missense | - | - | G64V | 9.7 | Benign | No |
| *TRIP4* | Medium | 15 | 64737241 ^d^ | G | A | Missense | - | - | V538I | 23.2 | Benign | No |
| *RBBP6* | Medium | 16 | 24583228 ^d^ | C | T | Missense | - | - | P1614L | 23.6 | Benign | No |
| *IL27* | Medium | 16 | 28511206 | C | G | Missense | rs147413292 | 6.3% | E166D | 9.5 | Benign | No |
| *NOD2* | Medium | 16 | 50745926 ^d^ | C | T | Missense | rs2066844 | 2.27% | R702W | 24.6 | Possibly damaging | Yes |
| *RBL2* | Medium | 16 | 53515590 ^d^ | C | T | Missense | - | - | A1031V | 19.3 | Benign | No |
| *COX4I1* | Medium | 16 | 85838545 | A | G | Missense | rs147346083 | 0.02% | S26G | 14.9 | Benign | No |
| *TYK2* | Medium | 19 | 10475649 | C | T | Missense | rs2304255 | 6.4% | G363S | 0.11 | Benign | No |
| *LRRC25* | Medium | 19 | 18507073 ^d^ | G | A | Missense | - | - | P234L | 1.9 | Benign | No |
| *BCKDHA* | - | 19 | 41916587 ^d^ | C | T | Missense | - | - | P52S | 23 | Benign | No |
| *SCAF1* | Medium | 19 | 50156323 | G | A | Missense | - | - | G893S | 22.9 | Benign | No |
| *SCAF1* | Medium | 19 | 50156330 | C | A | Missense | - | - | T895N | 12.8 | Benign | No |
| *ISOC2* | Medium | 19 | 55964727 ^d^ | G | - | Frameshift deletion | - | - | P119fs | 34 | - | Yes |
| *ZIM2* | Medium | 19 | 57301244 ^d^ | G | A | Missense | - | - | S158F | 6.164 | Benign | No |
| *NSFL1C* | Medium | 20 | 1434931 ^d^ | T | A | Missense | - | - | Y155F | 28.3 | Probably damaging | Yes |
| *CRELD2* | Medium | 22 | 50315340 ^d^ | C | T | Missense | - | - | P175S | 0.203 | Benign | No |
| **Approach #7: Dominant model - Early-onset cases in the family**  **(*de novo* variant: only the twins are heterozygous for the variant).** | | | | | | | | | | | | |
| *NUP153* | High | 6 | 17625023 ^d^ | C | A | Missense | - | - | A1315S | 23.9 | Possibly damaging | No |
| AA: amino acid; Alt: Alternative allele; CADD: Combined annotation dependent depletion; Chr: Chromosome, ExAC: Exome Aggregation Consortium; GDI: Gene damage index; MAF: Minor allele frequency; Ref: Reference allele. | | | | | | | | | | | | |
| **a** dbSNP (2016), GDI (January 2016), CADD v1.4 and PolyPhen-2 v2.2.2r398. | | | | | | | | | | | | |
| **b** Genomic position on GRCh37. | | | | | | | | | | | | |
| **c** Candidate variants were prioritized if they presented all the three following criteria: i) the variant had CADD ≥ 20, ii) the variant was a predicted damaging missense variant (PolyPhen-2), nonsense variant or frameshift indel and iii) the variant was located in a gene with low/medium GDI score. In approach #4, variants were prioritized if the two or more compound heterozygous variants reached criteria i and ii, while the gene reached criteria iii. Prioritized variants are highlighted in yellow in the table.  **d** Genotypes from WGS were validated by WES. | | | | | | | | | | | | |
| * The twin's aunt also carries the same mutation in this gene as the affected members of the family. | | | | | | | | | | | | |
