# Supplementary material for: Allele-dependent interaction of LRRK2 and NOD2 in leprosy
Source: PLoS Pathog. 2023 Mar 27;19(3):e1011260. doi: 10.1371/journal.ppat.1011260 (PMC10079233; doi:10.1371/journal.ppat.1011260)
Supplement: S3 Table — (DOCX) [file ppat.1011260.s010.docx]

**S3 Table.** Candidate deletion structural variants (DSVs) identified in the studied family by applying the custom filtering approaches shown in S1 Fig.

| **Chr** | **Start**** | **End**** | **Length (Kb)** | **Gene** | **Gene overlap** | **SV overlap^#^** | **Observed loss^¶^** |
| --- | --- | --- | --- | --- | --- | --- | --- |
| **Approach #5: Dominant model - All cases in the family, regardless of age-at-diagnosis**  **(Grandmother, father and twins are heterozygous for the variant).** | | | | | | | |
| **5** | 132918958 | 132925014 | 6.06 | *FSTL4* | CDS | esv2663891 | 398/1151 |
| **18** | 46997685 | 47005333 | 7.65 | *C18orf32* | EXON | esv3642494 | 203/2504 |
| **22** | 44564926 | 44566040 | 1.11 | *PARVB* | EXON | esv3647889 | 213/2504 |
| **Approach #6: Dominant model - Younger cases in the family**  **(Father and twins are heterozygous for the variant).** | | | | | | | |
| **9** | 132373830 | 132374787 | 0.96 | *C9orf50* | CDS | esv3621837 | 14/2504 |
| **Genomic position on GRCh37. | | | | | | | |
| ^#^ Accession number of structural variant detected in The 1000 genomes consortium that overlaps with the DSV in this study. | | | | | | | |
| ^¶^ Number of samples with SV (homozygous + heterozygous)/ Total number of samples sequenced by The 1000 genomes consortium. | | | | | | | |
