# Supplementary material for: Allele-dependent interaction of LRRK2 and NOD2 in leprosy
Source: PLoS Pathog. 2023 Mar 27;19(3):e1011260. doi: 10.1371/journal.ppat.1011260 (PMC10079233; doi:10.1371/journal.ppat.1011260)
Supplement: S4 Table — (DOCX) [file ppat.1011260.s011.docx]

**S4 Table.** Minor allele frequency (MAF) in GnomAD super-populations and estimated genotype frequencies of the *LRRK2* and *NOD2* variants found in the twins.

| **Population (GnomAD)** | **MAF (GnomAD)** | | | **Estimated genotype frequency**  **(Assuming complete LD between N551K & R1398H)** | | | |
| --- | --- | --- | --- | --- | --- | --- | --- |
|  | ***LRRK2* N551K** | ***LRRK2* R1398H** | ***NOD2* R702W** | **Homozygous N551K/R1398H** | **Heterozygous R702W** | **Homozygous R1398H & Heterozygous R702W** | |
| **Latino/Admixed American** | **16.2%** | **14.7%** | **2.1%** | **2.1%-2.6%** | **4.0%** | **0.09%-0.10%** | |
| European (Non-Finnish) | 7.2% | 7.2% | 4.3% | 0.5% | 8.2% | 0.04% | |
| Ashkenazi Jewish | 9.5% | 9.4% | 2.2% | 0.9% | 4.4% | 0.04% | |
| **African/African American** | **14.2%** | **14.0%** | **0.7%** | **2.0%** | **1.4%** | **0.03%** | |
| Other | 7.7% | 7.7% | 3.0% | 0.6% | 5.8% | 0.03% | |
| European (Finnish) | 5.7% | 5.6% | 1.8% | 0.3% | 3.6% | 0.01% | |
| South Asian | 4.0% | 4.0% | 0.04% | 0.2% | 0.1% | 0.0001% | |
| East Asian | 10.3% | 9.8% | 0% | 1.0%-1.1% | 0% | 0% | |
| Global | 8.7% | 8.5% | 2.6% | 0.7%-0.8% | 5.0% | 0.04% | |
| *** In bold:** closest populations from the studied family based on PCA. | | | | | | |  |
