# Supplementary material for: Allele-dependent interaction of LRRK2 and NOD2 in leprosy
Source: PLoS Pathog. 2023 Mar 27;19(3):e1011260. doi: 10.1371/journal.ppat.1011260 (PMC10079233; doi:10.1371/journal.ppat.1011260)
Supplement: S5 Table — (DOCX) [file ppat.1011260.s012.docx]

**S5 Table.** Genes previously associated with leprosy in GWAS or target association studies up to January 2023 that were included in the leprosy gene analysis from the WGS data (S1 Fig).

| **Chromosome** | **Known leprosy genes** |
| --- | --- |
| chr1 | *BATF3, BCL10, CFH, FLG, IL10, IL12RB2, IL23R, MASP2, PKLR, PTPN22* |
| chr2 | *IL18R1, IL18RAP, IL1RL1, IL1RN, MRPS5, SLC11A1* |
| chr3 | *IRAK2, MASP1, NCKIPSD, PPARG, SYN2* |
| chr4 | *TLR1, TLR2* |
| chr5 | *CDH18, IL12B, MIR146A* |
| chr6 | *C4B, C6orf15, CDSN, HLA-A, HLA-B, HLA-C, HLA-DPB1, HLA-DQA1, HLA-DQB1, HLA-DRB1, LTA, MICA, MICB, PARK2, PRKN, PSORS1C1, RAB32, SOD2, TNF* |
| chr7 | *ADAP1, BBS9* |
| chr8 | *CTSB, MED30, RIPK2, SLC7A2* |
| chr9 | *CARD9, DEC1, FCN2, MIRLET7A1, TLR4, TNFSF15, TNFSF8* |
| chr10 | *ADO, EGR2, GATA3, MBL2, MRC1, SLC29A3* |
| chr11 | *CCDC88B* |
| chr12 | *ALDH2, GIT2, IFNG, LRRK2, LTA4H, P2RX7, VDR* |
| chr13 | *CCDC122, LACC1* |
| chr14 | *HIF1A, ZFP36L1* |
| chr16 | *CIITA, COX4I1, CYLD, IL27, NOD2, RMI2* |
| chr17 | *ERBB2* |
| chr19 | *SIGLEC5, TYK2* |
| chr22 | *TCN2* |
