# Supplementary material for: Allele-dependent interaction of LRRK2 and NOD2 in leprosy
Source: PLoS Pathog. 2023 Mar 27;19(3):e1011260. doi: 10.1371/journal.ppat.1011260 (PMC10079233; doi:10.1371/journal.ppat.1011260)
Supplement: S6 Table — (DOCX) [file ppat.1011260.s013.docx]

**S6 Table.** Oligonucleotides used in the CRISPR/Cas construct of *Lrrk2* variants.

| **Forward and reverse target DNA oligonucleotides** | **Sequences** |
| --- | --- |
| N551K forward | 5’-TAATACGACTCACTATAGGAACAGGGTATGTAGA-3’ |
| N551K reverse | 5’-TTCTAGCTCTAAAACTTGTTCTACATACCCTGTT-3’ |
| R1398H forward | 5’-TAATACGACTCACTATAGAGAATTCCTCACGACC-3’ |
| R1398H reverse | 5’-TTCTAGCTCTAAAACTCTAGGTCGTGAGGAATTC-3’ |
| **Donor HDR templates** | **Sequences** |
| N551K | 5’A*G*T*AAACAACAAGAAAGTAAAGTAAAACTCAAAGCCCCA |
|  | CCCCCAGACCCTCAGATGTTAGTCTCGTTAATGGTATAAAGA |
|  | CAGAAAATCCTTGTTCTACATACCCTCTTCAGAGCGACTAGA |
|  | ACCAGCTTGTGGATGTCAGTCCTGAAACACTGTTTTCTGAGC |
|  | ACATTTGGTCTGCATTGAGA*G*T*C-3’ |
| R1398H | 5’-A*G*A*TCATAGACAGCCAGGTAGAGGGCTCTCTGGGTCAT |
|  | GAAGTGCGGGTGAGTGCTGTAGAATTCCTCATGACCTAGA |
|  | AGGAGATATCAGAGGTTTGAGTCTTTCCCATAGTAGGTAGG |
|  | ACTCGTTACGAAATAAT*G*A*G-3’ |
| **Sequences of PCR primers** | **Sequences** |
| N551K forward | 5’-GGCAGTGTGTGGAGCCTAAA-3’ |
| N551K reverse | 5’-GGCATCAGAGAAGACAGCCA-3’ |
| R1398H forward | 5’-GTCCATCCAAATACGGGGCA-3’ |
| R1398H reverse | 5’-GGGCATCCAGGGACACATAA-3’ |
| *Phosphorothioate modification | |
