# Supplementary material for: Allele-dependent interaction of LRRK2 and NOD2 in leprosy
Source: PLoS Pathog. 2023 Mar 27;19(3):e1011260. doi: 10.1371/journal.ppat.1011260 (PMC10079233; doi:10.1371/journal.ppat.1011260)
Supplement: S1 Method — (DOCX) [file ppat.1011260.s015.docx]

**S1 Methods. Whole exome sequencing for variant validation.**

In addition to the WGS, we had whole exome sequencing (WES) data available for five family members (ID2 and D4 to ID7 from Fig 1B). As the WES was generated with a different technology than WGS (WES: Ion Proton Sequencer, Thermo Fisher Scientific; WGS: HiSeq 2500, Illumina), it was used as an independent genotype confirmation of the candidate SNVs and short Indels detected by the WGS analysis (S2 Table). Exome was captured using the Targetseq Exome kit (Thermo Fisher Scientific). To cover the target regions, this in-solution array contains more than 2 million oligonucleotide probes ranging from 60 to 100 bp that tile 52.7 Mb of target regions including the exome and flanking areas. Then, the exon-enriched DNA libraries were sequenced by 200 bp single-end reads on Ion Proton Sequencer using the Ion PI Chip v2 (Thermo Fisher Scientific). Quality assessment of the raw data was performed using FastQC software. Sequence data analysis was conducted using a pipeline for variant discovery with Torrent Suite (TS) software v5.0 available on GitHub (https://github.com/iontorrent/TS). Using TS, reads were aligned to the human reference using map4 command line implemented on TMAP (https://github.com/iontorrent/TS/tree/master/Analysis/TMAP). Mapped reads were sorted according to their genomic coordinate position using SortOrder command and PCR duplicates were flagged with MarkDuplicates in Picard. Quality assessment of the mapped reads was performed using QualiMap. Variant calling was performed with Torrent variant caller (TVC) plugin from TS software, using “Germline - Proton TargetSeq - High stringency” parameter option with default settings (https://github.com/iontorrent/TS/tree/master/plugin/variantCaller). Identification of single nucleotide variants (SNV) and dinucleotide variants (DNV) was performed in regions with coverage ≥ 10X, while indel calling was performed only in regions with coverage ≥ 20X. The lists of variants from all the samples were combined in one multi-samples VCF file using CombineVariants tool in GATK. Finally, genotypes of the candidate variants from the WGS filtering approaches were compared between WGS and WES for variants detected by both methods (S2 Table).
